# Exploring the relationship between breastfeeding and the incidence of infant illnesses in Ireland: evidence from a nationally representative prospective cohort study

**DOI:** 10.1186/s12889-023-15045-8

**Published:** 2023-01-20

**Authors:** Sarah Murphy, Laura Carter, Tasneem Al Shizawi, Michelle Queally, Sarah Brennan, Stephen O’Neill

**Affiliations:** 1 School of Medicine, University of Galway, Galway, Ireland; 2 J.E. Cairnes School of Business and Economics, University of Galway, Galway, Ireland; 3 Department of Enterprise and Technology, Atlantic Technological University, Galway, Ireland; 4 Department of General Practice & Donegal Medical Academy, University of Galway, Galway, Ireland; 5 General Practice, Carrigart Health Centre, Carrigart, Carrigart, Co. Donegal, Ireland; 6 Department of Health Services Research and Policy, London School of Hygiene and Tropical Medicine, London, UK; 7 Centre for Economic and Social Research on Dementia, University of Galway, Galway, Ireland

**Keywords:** Breastfeeding, Infant illness, Weighting, Propensity score matching, Entropy balancing

## Abstract

**Background:**

Ireland has one of the lowest BF rates in the world. This study investigates the association between breastfeeding and infant health in Ireland.

**Methods:**

A cross-sectional, secondary analysis of data collected from *Growing Up in Ireland* (GUI): *the National Longitudinal Study of Children* was conducted. The average morbidity for 2212.

infants exclusively breastfed for at least 90 days (EBF90days) was compared to data for 3987 infants in the non-breastfed (Non-BF) group. Data were weighted using entropy balancing to ensure the comparability of groups. Sensitivity analyses considered alternative definitions of the breastfeeding group.

**Results:**

Infants who were EBF90days were significantly less likely to be admitted to hospital (CI: − 0.06 to − 0.03), spent less nights in hospital (CI: − 0.37 to − 0.11), and were less likely to develop respiratory diseases including asthma (CI: − 0.03 to − 0.01), chest infections (CI: − 0.12 to − 0.08), snuffles/common colds (CI: − 0.07 to − 0.02), ear infections (CI: − 0.08 to − 0.04), eczema (CI: − 0.08 to − 0.04), skin problems (CI: − 0.04 to − 0.00), wheezing or asthma (CI: − 0.06 to − 0.03), vomiting (CI: − 0.03 to − 0.00), and colic (CI: − 0.04 to − 0.01). Further outcomes such as current health of the infant at time of interview (CI: − 0.04 to − 0.00), feeding problems (CI: − 0.04 to − 0.02) and sleeping problems (CI: − 0.02 to − 0.00) indicated a protective effect of EBF90days versus Non-BF. However, these infants were also more likely to fail to gain weight (CI: 0.01 to 0.02) and were at a slightly higher risk of developing nappy rash (CI: 0.00 to 0.02).

**Conclusion:**

Exclusive breastfeeding for 90+ days is associated with protection against childhood morbidity. Given the protective effect of breastfeeding on adverse health effects in infants, policy makers should prioritise policies that support, promote and protect exclusive breastfeeding.

**Supplementary Information:**

The online version contains supplementary material available at 10.1186/s12889-023-15045-8.

## Background

By 2025, the World Health Organization (WHO) aims to achieve a 50% universal exclusive breastfeeding (EBF) rate in the first 6 months which is expected to significantly reduce maternal, neonatal, infant and childhood mortality [1]. In Ireland, poor practice of EBF prevails, with only 15% of children exclusively breastfed for the first 6 months compared with the global average of 38% and European average of 25% [2]. The WHO has identified several factors which contribute to a low rate of exclusive breastfeeding, including knowledge-related factors [3].

Human milk has long been believed to protect against infection in infants [4, 5]. A vast scientific literature demonstrates the substantial health, social and economic importance of breastfeeding, including lower infant and young child morbidity and mortality from diarrhoea and other infectious diseases [6, 7]. The health benefits for mothers who breastfeed include a reduced risk of breast, ovarian, cervical and endometrial cancers, as well as reduced risk of anaemia and protection against osteoporosis and hip fracture [8, 9]. Given the health benefits of breastfeeding for both mother and child, the WHO recommend that infants ought to be exclusively breastfed for the first 6 months of life and that breastfeeding should continue as part of their diet with appropriate complementary weaning foods up to 2 years old and beyond [10]. However, despite gradual increases over the last ten years, Ireland’s breastfeeding rates continue to be the lowest in international comparisons [11] with implications for maternal and child health.

To our knowledge, no studies to date have explored the relationship between breastfeeding and the incidence of infant illnesses in Ireland. This knowledge gap is compounded by the fact that no coordinated national breastfeeding monitoring system exists beyond the point of hospital discharge in Ireland, resulting in a lack of knowledge regarding national breastfeeding data. Ireland does not yet report to the World Breastfeeding Trends Initiative (WBTi) which collates data on the degree of implementation of the *Global Strategy for Infant and Young Child Feeding* [12]. Country-specific data on the relationships between breastfeeding practices and infant illnesses can inform policies supportive of early, exclusive and extended breastfeeding practises within population specific promotion programmes.

The literature relating to breastfeeding and infant illness incidence almost exclusively relies on evidence from observational studies. Research suggests that breastfeeding is linked with infant health benefits [13]. There is also evidence however that the benefits are overstated due to selection bias [14, 15]. Mothers that self-select into breastfeeding rather than formula feeding may differ from those that do not in ways that influence infant health [16]. Without accounting for baseline maternal differences in the research design or fully including all confounding variables, statistical models may tend to overstate the positive relationship between breastfeeding and infant health.

The objective of this study is to investigate the relationship between exclusive breastfeeding for at least 90 days and the incidence of infant illnesses in an Irish cohort, while accounting for such self-selection through weighting. We hypothesise that in an Irish infant cohort with high breast milk substitute use, morbidity among infants aged 0–9 months will be significantly lower in the exclusively breastfed cohort.

## Methods

### Data

Data were obtained from the first wave of the Growing Up in Ireland (GUI) survey, a longitudinal cohort study of a nationally representative sample of over 11,000 infants. The GUI eligibility criteria for wave one was that the infant must be nine-months of age at the time the survey was conducted, between the beginning of September 2008 to the end of April 2009. The infants were randomly selected from the Child Benefit Register which recorded 41,185 eligible births between 1st December 2007 and 30th June 2008. The valid contact response rate was very high (70.2%).

The GUI survey, asked parents whether the infant had been taken to a General practitioners (GP), Health Centre or Health visitor, or to Accident and Emergency for a range of conditions including whether the infant suffered from: cold, chest infections, ear infections, respiratory illness, digestive allergies, eczema, kidney disease, asthma, vomiting, diarrhoea/ constipation, meningitis, colic skin problems, nappy rash, failure to grow, developmental delay, feeding problems, sleeping problems, or dental problems. The survey asked about the health of the infant at birth, the current health of the infant, the severity of the infant’s most severe illness (minor, moderate or severe). The parents were also asked to report the number of times the infant was admitted to hospital and the average number of nights spent by the infant in hospital.

GUI records information on whether the infant was currently breastfed or the age at which breastfeeding stopped as well as additional questions regarding whether the infant was ever exclusively breastfed, was still exclusively breastfed or the age when exclusive breastfeeding stopped. Considering the numbers of infants reported to be exclusively breastfed at 90 and 180 days were 2212 and 712 respectively, in our primary analysis we focus on a comparison between infants that were exclusively breastfed for at least 90 days (EBF90days) and those that were never breastfed (Non-BF) thus receiving only formula from birth given the greater statistical power to detect effects. In the appendix, we also conduct sensitivity analyses (SA) by considering three further comparisons: (SA1) between infants that were ever breastfed (BF) and those that were never breastfed (Non-BF) and (SA2) between infants that were exclusively breastfed (EBF) for any length of time and those not exclusively breastfed (non-EBF) i.e. receiving any formula from birth and (SA3) between infants that were exclusively BF for at least 90 days (EBF90days) and compare these to the non-EBF group.

We assign infants to the relevant groups based on the respondent’s responses to the question “Was <baby> ever breastfed?”, and “was <baby> ever exclusively breastfed”, and “How old was <baby> when he/she stopped being exclusively breastfed?”

Unfortunately, while response rates were generally high, data on at least one of the above-mentioned variables was unavailable for 1255 infants, resulting in a final analysis sample of 9879 infants for whom complete information was available (see Appendix for details and Fig. A12). As can be seen in Table A1 in the appendix, the rate of missingness was similar across our comparison groups, suggesting our assumption that data is ‘missing at random’ is plausible in this context.

### Ethical approval

Ethical approval for this study was obtained through the College of Medicine, Nursing and Health Sciences Research Ethics Committee at the National University of Ireland Galway in March 2021.

### Data analysis

A naïve comparison could be made by comparing the outcomes (e.g., incidence of each illness) between the two groups (EBF90days vs Non-BF). A concern with such an approach is that the compositions of the groups could differ e.g. it may be the case the infants born into higher socioeconomic groups may be more likely to be exclusively breastfed for at least 90 days, [17] leading us to wrongly attribute the effects of such variables on outcomes to be an effect of the fact the infant was EBF90days. Regression adjustment can be used to control for such covariates to improve the reliability of comparisons. However, it can be challenging to correctly specify regression models. Methods such as propensity score matching (PSM) [18] and inverse probability weighting (IPW) [19, 20] can be used to reduce the risk of bias from model mis-specification by making the ‘treated’ and ‘control’ groups similar in terms of their covariates.

In this study we use Entropy Balancing [21] which extends inverse probability weighting methods by balancing the covariate moments (mean, variance and skewness for continuous variables, and mean for binary variables) of the comparison groups, while choosing weights that are as close as possible to uniformly distributed – reducing the tendency of IPW methods to give extremely high weights to some individuals. Entropy Balancing has been shown to be doubly robust [22] meaning that provided either the linear outcome regression or the logistic propensity score model implicitly underlying estimates are correctly specified, estimates will be unbiased. Differences in outcomes with and without exclusive breast feeding for 90 days can be estimated using separate weighted regressions with each outcome of interest as the dependent variable (*Y*_*i*_) and an indicator for the group of interest (*D*_*i*_= 1 if EBF90days, 0 if Non-BF), using the entropy balancing weights to control for differences in covariates between the groups. The regression model is specified as:

$${Y}_i={\boldsymbol{X}}_i\boldsymbol{\beta} +\tau {D}_i+{\epsilon}_i$$

where ***X***_*i*_ is a vector of their covariates, with coefficients denoted by ***β*** and *ϵ*_*i*_ captures idiosyncratic shocks and individuals are weighted by the entropy balancing weights. The coefficient on *D*_*i*_ captures the difference in conditional means between the two groups, and is our parameter of interest. Controlling for observed covariates is not essential here since entropy balancing tends to achieve near perfect covariate balance, hence we exclude ***X***_*i*_.

The observed potential confounders to control for were informed by data availability and an extensive literature review. We control for an extensive set of variables (see appendix Table A2 for a full list of covariates) that can be summarised under the following headings: health of the infant at birth, the antenatal care received, pregnancy complications, folic acid consumption, maternal smoking history, method of delivery, stage of gestation at which the infant was born, infant’s weight at birth, birth complications, household equivalent annual income, highest education received by mother, hours’ sleep infant receives, and whether or not the infant has received their vaccinations.

## Results

### Covariate balance

Our estimation sample size was 9879 infants following exclusion of those who were missing these variables of interest. Tables A2 to A5 in the appendix compare the means of each group before and after reweighting and report the absolute standardised differences between the groups for our 4 comparisons respectively, including the socio-economic and demographic background of the mothers**.** A standardized difference greater than 0.10 is indicative of imbalance [23]. In general, we find balance is relatively good even before adjustment, while weighting by the entropy balancing weights almost completely eliminates imbalance. Thus, comparisons between groups after weighting should not be susceptible to observed confounding.

### Comparisons of outcomes between (weighted) groups

Descriptive statistics for the sample are reported in Table A9. There were 2212 infants that were EBF for at least 90 days compared to 3987 Non-BF babies. In Table 1, we compared the outcomes for EBF90days and Non-BF infants after reweighting the Non-BF group using entropy balancing weights to ensure the groups are comparable in terms of observed covariates (see appendix Table A2). The absolute risk difference between the EBF90days and Non-BF groups at 9 months in the incidence of chest infection (− 0.10 (CI: − 0.12 to − 0.08)), snuffles/common colds (− 0.05 (CI: − 0.07 to − 0.02)), ear infections (− 0.06 (CI:-0.08 to − 0.04)), asthmatic symptoms (− 0.05 (CI: − 0.06 to − 0.03)), respiratory symptoms (− 0.02 (CI: − 0.03 to − 0.01)), eczema or skin allergies (− 0.03 (CI: − 0.05 to − 0.02)), skin problems (− 0.02 (CI: − 0.04 to − 0.00)), vomiting (− 0.02 (CI: − 0.03 to − 0.00)) and colic (− 0.02 (CI: − 0.04 to − 0.01)) indicated a protective effect of EBF90days versus Non-BF. Further statistically significant protective effects were obtained for outcomes such as current health of the infant at time of interview (− 0.02 (CI: − 0.04 to − 0.00)), feeding problems (− 0.03 (CI: − 0.04 to − 0.02)) and sleeping problems (− 0.01 (CI: − 0.02 to − 0.00).

**Table 1:** Difference in outcomes for EBF (90+ days) versus Non-BF after weighting to ensure covariate balance using Entropy Balancing weights

	EBF90days versus Non-BF Difference	95% Confidence interval
Average number of nights spent in hospital by baby	−0.240	(− 0.373, − 0.107)
Current health of the baby	− 0.024	(− 0.044, − 0.004)
Baby admitted to hospital	− 0.043	(− 0.059, − 0.028)
Respiratory disease [including asthma]	− 0.022	(− 0.031, − 0.013)
Digestive allergies (e.g. lactose intolerant)	−0.004	(− 0.013, 0.006)
Eczema or any kind of skin allergy	−0.033	(− 0.049, − 0.016)
Kidney disease	− 0.002	(− 0.006, 0.002)
Any developmental delay	− 0.004	(− 0.008, 0.001)
Snuffles/common cold	− 0.045	(− 0.070, − 0.021)
Chest infection	− 0.100	(− 0.123, − 0.078)
Ear infection	−0.058	(− 0.077, − 0.040)
Feeding problems	− 0.031	(− 0.043, − 0.019)
Sleeping problems	−0.011	(− 0.019, − 0.003)
Dental problems	0.005	(− 0.003, 0.013)
Wheezing or asthma	−0.045	(− 0.058, − 0.032)
Skin problem	− 0.020	(− 0.038, − 0.002)
Persistent nappy rash	0.009	(0.001, 0.017)
Failure to gain weight or to grow	0.013	(0.006, 0.020)
Persistent vomiting	−0.015	(−0.025, − 0.004)
Persistent diarrhoea/Constipation	− 0.002	(− 0.013, 0.010)
Meningitis	0.000	(−0.002, 0.003)
Colic	−0.023	(−0.035, − 0.011)

Number of observations 6199.

EBF90days was also associated with lower health care resource use, with an absolute risk reduction for being admitted to hospital (− 0.04 (CI: − 0.06 to − 0.03)) and a reduction in nights spent in hospital (− 0.24 (CI: − 0.37 to − 0.11)). However, EBF90days was associated with an increase in the absolute risk of failure to grow (0.01 (CI: 0.01 to 0.02)) and persistent nappy rash (0.01 (CI: 0.00 to 0.02)). Table A6 in the appendix, shows the results of three further comparisons (a), (b) and (c) followed a similar pattern to those reported for the EBF90days versus Non-BF comparison.

### Comparisons of outcomes between (weighted) groups after standardisation

**Fig. 1:**
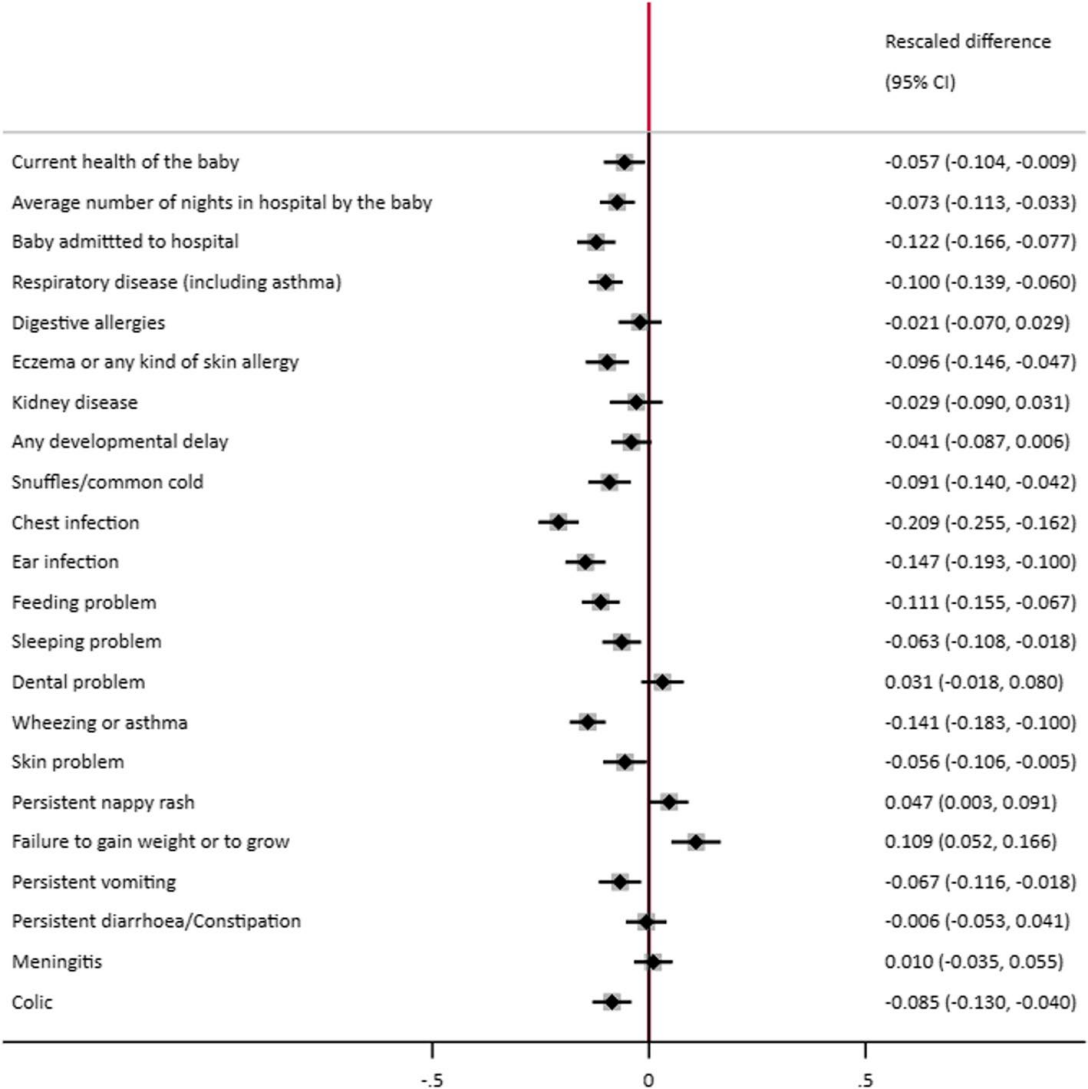
Entropy Balanced comparison of outcomes for EBF (90+ days) versus Non-BF rescaled by Non-BF standard deviation

Since there are many outcomes and these are measured on different scales, in Fig. 1 we present a forest plot of the estimates after rescaling the difference in outcome between groups by the standard deviation of the comparison group for each outcome. Comparisons between the EBF180 and NBF groups show similar magnitudes of changes, with wider confidence intervals reflecting the lower numbers in the EBF180 groups. Figures A1 to A3 in the appendix, present forest plots for our three additional comparisons (SA1), (SA2) and (SA3). Generally, across all comparisons the magnitudes of the differences are relatively small when expressed in terms of the standard deviation of the outcome, nonetheless at the population level these could translate into substantial differences.

### Sensitivity analyses

Results based on naïve unadjusted comparisons (see appendix Table A7, Figs. A4 to A7) or using PSM (see appendix Table A8, Figs. A8 to A11) in place of entropy balancing are presented in the appendices and illustrate that results are not very sensitive to the choice of analytic approach, although the propensity score matched estimates yield less statistically significant differences as this approach is less efficient given that it does not use all of the available data unlike the other approaches.

## Discussion

Breastfeeding has not yet reached optimal prevalence in many countries, including Ireland [2, 5]. With a formula feeding rate of 43.1% in 2016, Ireland is a fertile population in which to study associated effects of infant feeding types on infant morbidity. The objective of this paper was to investigate the relationship between exclusive breastfeeding for at least 90 days and the incidence of infant illness while controlling for a broad range of potential confounders in an Irish cohort. We find that infants who were EBF for 90+ days were significantly less likely to be admitted to hospital, spent less nights in hospital, and were less likely to develop respiratory diseases including asthma, snuffles/common colds, chest infections, eczema, ear infections, wheezing and asthma, skin problems, vomiting, and colic indicating a protective effect of breastfeeding. Further outcomes such as current health of the infant at time of interview, feeding problems and sleeping problems were also statistically significantly negative, signalling the potential protective effect of EBF90days. However, these infants were also more likely to fail to gain weight and to develop nappy rash. It should be noted however that the implied differences with and without exclusive breast feeding for 90 plus days tend to be relatively small compared to the inherent variability of the outcome, less than 0.15 standard deviations in all cases (Fig. 1). The results of this research conforms to the international literature [24, 25]. But are striking in that they show that even in a high income country such as Ireland, breastfeeding is correlated with infant morbidity and health care utilisation.

Our estimates indicate that, given the population of Irish infants born in the year GUI was collected (2008) was 73,996 [26], there would have been 17,766 less nights spent in hospital if all infants were exclusively breastfed for at least 90 days versus had none of them been breastfed. Furthermore, there would have been 1644 fewer cases of respiratory illnesses, 7429 fewer cases of chest infection and 4320 fewer cases of ear infections. This data again points to the risks of early introduction of formula into the diet of a breastfed baby and supports exclusive breastfeeding for at least 90 days and termination of the practice of human milk substitute supplementation for reasons avoidable by proper planning, antenatal expression of colostrum [27, 28] and availability of donor milk from community milk banks [29]. These practices are envisaged in the recent Code of Marketing of Breast Milk Substitutes’ policy adopted by the HSE [30] and WHO/UNICEF’s 10 steps to successful breastfeeding [31].

The study reports a statistically significant increase in failure to grow among the cohort that were EBF for at least 90 days. In 2013, the Health Service Executive (HSE) in Ireland, introduced the use of new WHO growth charts, which were developed using natural infant weight gains in breastfeeding cohorts and thus showed slower weight gain trajectory in infants than in the previously used HSE growth charts developed using ‘natural’ weight gain data from formula fed cohorts. The use of the newer WHO growth charts is recommended in order to avoid mislabelling infants as underweight or failing to thrive [24] which led to unnecessary supplementation and cessation of breastfeeding. However, during the study period in 2008 Irish health care professionals were still using the old HSE growth charts which potentially led to erroneous beliefs among parents regarding failure to thrive/gain weight. Thus, parents of breastfed infants may have been more likely to report that their children exhibited ‘Failure to gain weight or to grow’. Previous research analysing GUI data has shown a significant risk reduction for obesity development in BF infants, with this risk reduction being greater with increasing duration of breastfeeding [32].

Our analysis also showed that infants in the EBF90days group were more commonly reported to have persistent nappy rash (0.01 (CI: 0.00 to 0.02)) in comparison to the Non-BF cohort. A similar finding has also been observed in the 1997 Avon longitudinal dataset analysis of over 12,000 infants, which reported that breastfeeding is a risk factor for the development of nappy rash [33] in the first 4 weeks of life. This study also found frequent stooling, a phenomena associated with breastfeeding, to be an associated risk factor of diaper dermatitis. Despite this, studies show a protective effect of breastfeeding on nappy rash over the first year of life [34]. This finding in our study may relate to the presence of more frequent stooling, as is normal in exclusively breastfed infants and thus in the EBF90days cohort, who are similarly aged to those participants in the Avon study. This frequent stooling in exclusively fed infants is explained by the presence of Human Milk Oligosaccharides (HMOs), prebiotics for gastrointestinal microorganisms which, as indigestible by infants, leads to an osmotic laxative effect [35]. The whey dominant whey:casein ratio in breastmilk, in comparison to a casein dominant ratio in formula, also contributes to increased stooling in exclusively breastfed infants [36].

Another consideration is the culturally accepted use of commercial infant wipes to clean babies on napkin changing. There is evidence to show that frequent use of multi-ingredient baby wipes leads to increased incidence of napkin rash [37]. As the cohort of infants in the GUI is from 2008, participants would have used wipes containing multiple ingredients rather than those subsequently invented with only 2 ingredients which were shown to be associated with a lower incidence of napkin dermatitis [37]. Interestingly, a case control study, albeit with only 30 participants, also has shown statistically significant therapeutic impact of breastmilk on napkin rashes [38]. As napkin dermatitis results in significant difficulty for both parents and infants, this area would warrant further research considering that an effective free easily assessed therapeutic option, such as breastmilk, could help significantly reduce this suffering.

The findings from this study conform with the international data showing that the incidence of infectious illnesses increases with increasing exposure to human milk substitutes and that exclusive human milk feeding is most protective in terms of infectious illnesses [4, 5, 8, 11, 25]. Our results indicate that the protective effect against developing at least 1 of 4 infectious diseases examined, namely, chest infection, ear infection, common colds and meningitis; in a cohort of 100 infants EBF for 90, we estimate a reduction of 9.3 infants [CI 11.7 to 6.9] presenting with one of these infective illnesses when EBF for at least 90 days compared to a similar formula fed [Non-BF] cohort. Similar point estimates were found in the EBF180 vs NBF analysis, indicating beneficial effect of exclusive breastfeeding to 180 days as recommended by the WHO, albeit there was lower power to detect effects due to the reduced sample sizes for that analysis.

Furthermore, our study shows greater protection may be provided by EBF over non-EBF and Non-BF infants (see appendix Table A6) which leads us to consider that it is the risk of feeding human milk substitutes, rather than on the benefits of breastfeeding per se, that leads to differences in morbidity and health care utilisation, between the EBF, BF and Non-BF groups. This is not a new idea [39] but does emphasise the newer narrative that breastfeeding is the physiological normal, one which has driven mammalian evolution for millions of years [40] and that replacing human milk feeding with substitute human milks may infer risk. Our findings indicate an increased associated risk of morbidity with the early introduction of and substitution with human milk substitutes, with the risks greater among formula fed [Non-BF] infants.

There are some limitations to the present study. Most notably, GUI relies on recollection of whether a mother breastfed or not. However, there is evidence that maternal recollection of breastfeeding status tends to be a valid representation of breastfeeding status.^42^ We rely on self-reports of whether an infant was taken to the GP, Health Centre or Public Health Nurse or to Accident and Emergency for each of the conditions, which may be another source of misreporting bias. Linking the GUI data to administrative records was not possible in this study. Since GUI used a nationally representative sample, it is plausible that results would generalize to the population, although as we note in the appendix, results may be sensitive to the presence of unobserved confounders despite the rich set of covariates for which we controlled. Future work will explore the relationship between breastfeeding and the infants’ health in later waves. We could also assess the potential cost savings attributable to optimised breastfeeding in Ireland. Finally, we could explore whether findings are similar in other observational child cohort studies such as *Growing up in Scotland, Growing up in New Zealand* and *Growing up in Australia.*

## Conclusion

Research has shown that breastfeeding is the best source of infant nutrition and may prevent adverse health outcomes in infants. Findings from this study suggest that exclusive breastfeeding for at least 90 days is associated with protection against childhood morbidity and is also significantly associated with reduced health resource use in the form of hospital admission and reduced length of hospital stay. There is a need for research in this area to inform policy makers regarding the health benefits associated with breastfeeding and to provide an evidence base for appropriate funding of breastfeeding policy initiatives.

### Supplementary Information

**Additional file 1.**

**Additional file 2.**

**Additional file 3.**

## Data Availability

The datasets generated and/or analysed during the current study are available in the Irish Social Science Data Archive (ISSDA) repository: https://www.ucd.ie/issda/data/growingupinirelandgui/.

## Abbreviations

BF: Breastfed
CI: Confidence Interval
EBF: Exclusive Breastfeeding
EBF90days: Exclusive Breastfeeding for at least 90 days
GP: General Practitioner
GUI: Growing Up in Ireland
HMOs: Human Milk Oligosaccharides
HSE: Health Service Executive
IPW: Inverse Probability Weighting
Non-BF: Non-Breastfed
Non-EBF: Non-Exclusive Breastfeeding
PSM: Propensity Score Matching
WBTi: World Breastfeeding Trends Initiative
WHO: World Health Organization
