# Correction to: Exploring the relationship between breastfeeding and the incidence of infant illnesses in Ireland: evidence from a nationally representative prospective cohort study

**DOI:** 10.1186/s12889-023-15333-3

**Published:** 2023-10-10

**Authors:** Sarah Murphy, Laura Carter, Tasneem Al Shizawi, Michelle Queally, Sarah Brennan, Stephen O’Neill

**Affiliations:** 1 School of Medicine, University of Galway, Galway, Ireland; 2 J.E. Cairnes School of Business and Economics, University of Galway, Galway, Ireland; 3 Department of Enterprise and Technology, Atlantic Technological University, Galway, Ireland; 4 Department of General Practice & Donegal Medical Academy, University of Galway, Galway, Ireland; 5 General Practice, Carrigart Health Centre, Carrigart, Co. Donegal, Ireland; 6 Department of Health Services Research and Policy, London School of Hygiene and Tropical Medicine, London, UK; 7 Centre for Economic and Social Research on Dementia, University of Galway, Galway, Ireland

**Correction to:** ***BMC Public Health*** **23, 140 (2023)**

10.1186/s12889-023-15045-8

The original publication of this article was missing the following affiliation for author Stephen O’Neill:

- Centre for Economic and Social Research on Dementia, University of Galway, Galway, Ireland.

The original article has been updated to correct this.

