# Supplementary material for: Exploring the relationship between breastfeeding and the incidence of infant illnesses in Ireland: evidence from a nationally representative prospective cohort study
Source: BMC Public Health. 2023 Jan 20;23:140. doi: 10.1186/s12889-023-15045-8 (PMC9854149; doi:10.1186/s12889-023-15045-8)
Supplement: Supplementary file 1 — Additional file 1. [file 12889_2023_15045_MOESM1_ESM.docx]

**Table A1. Comparison of missingness across comparison groups**

|  | **Non-missing**  **N (%)** | **Missing**  **N (%)** |
| --- | --- | --- |
| **Exclusively Breastfed for at least 90 day (EBF90days)** | 2,212 (88.76%) | 280 (11.24%) |
| **Non-Breast fed (non-BF)** | 3,987 (90.26%) | 430 (9.74%) |
|  |  |  |
| **Other comparisons:** |  |  |
| **Breast fed (BF)** | 5,892 (87.76%) | 822 (12.24%) |
| **Non-Breast fed (non-BF)** | 3,987 (90.26%) | 430 (9.74%) |
|  |  |  |
| **Exclusively Breastfed (EBF)** | 4,716 (88.71%) | 606 (11.39%) |
| **Non-exclusively BF (non-EBF)** | 5,163 (88.91%) | 644 (11.09%) |
|  |  |  |
| **Exclusively Breastfed for at least 90 day (EBF90days)** | 2,212 (88.76%) | 280 (11.24%) |
| **Non-exclusively Breastfed for at least 90 day (non-EBF90days)** | 7,667 (88.71%) | 975 (11.28%) |

**Table A2. Imbalance in covariates by whether exclusively breastfed for at least 90 days or not breastfed**

|  | **EBF90days**  **Mean** | **Non-BF Mean** | **Std. diff.** | **EBF90days**  **Mean (weighted)** | **Non-BF Mean (weighted)** | **Std. diff.** |
| --- | --- | --- | --- | --- | --- | --- |
| Health at birth: Very healthy, no problems | 0.844 | 0.807 | 0.098 | 0.844 | 0.844 | 0.000 |
| Health at birth: Healthy, but a few minor problems | 0.133 | 0.163 | 0.084 | 0.133 | 0.133 | 0.000 |
| Health at birth: Sometimes quite ill | 0.014 | 0.018 | 0.030 | 0.014 | 0.014 | 0.000 |
| Health at birth: Almost always unwell | 0.008 | 0.012 | 0.034 | 0.008 | 0.008 | 0.000 |
| Antenatal care: Shared care (between GP and other professional) | 0.782 | 0.789 | 0.016 | 0.782 | 0.782 | 0.000 |
| Antenatal care: Private consultant alone | 0.134 | 0.104 | 0.092 | 0.134 | 0.134 | 0.000 |
| Antenatal care: Hospital clinic alone | 0.048 | 0.084 | 0.145 | 0.048 | 0.048 | 0.000 |
| Antenatal care: Midwives clinic alone | 0.027 | 0.018 | 0.060 | 0.027 | 0.027 | 0.000 |
| Antenatal care: Independent midwife alone | 0.008 | 0.002 | 0.096 | 0.008 | 0.008 | 0.000 |
| Antenatal care: Had no antenatal care | 0.001 | 0.004 | 0.059 | 0.001 | 0.001 | 0.000 |
| Pregnancy complication - Raised BP in isolation | 0.089 | 0.114 | 0.083 | 0.089 | 0.089 | 0.000 |
| Pregnancy complication - Pre-eclampsia | 0.040 | 0.088 | 0.196 | 0.040 | 0.040 | 0.000 |
| Pregnancy complication - Urinary/Kidney Infection | 0.097 | 0.181 | 0.246 | 0.097 | 0.097 | 0.000 |
| Pregnancy complication - Persistent Vomit/Nausea | 0.154 | 0.194 | 0.106 | 0.154 | 0.154 | 0.000 |
| Pregnancy complication - Gestational Diabetes, diet treated | 0.021 | 0.020 | 0.007 | 0.021 | 0.021 | 0.000 |
| Pregnancy complication - Gestational Diabetes, insulin treated | 0.008 | 0.011 | 0.032 | 0.008 | 0.008 | 0.000 |
| Pregnancy complication - Bleeding during Second Half Pregnancy | 0.046 | 0.066 | 0.091 | 0.046 | 0.046 | 0.000 |
| Pregnancy complication - Vaginal Infection during Pregnancy | 0.036 | 0.032 | 0.024 | 0.036 | 0.036 | 0.000 |
| Pregnancy complication - Intrauterine Growth Restriction (Small baby on scan) | 0.014 | 0.025 | 0.081 | 0.014 | 0.014 | 0.000 |
| Pregnancy complication - Rhesus Incompatibility | 0.034 | 0.038 | 0.018 | 0.034 | 0.034 | 0.000 |
| Pregnancy complication - Influenza | 0.037 | 0.040 | 0.013 | 0.037 | 0.037 | 0.000 |
| Pregnancy complication - Placenta Praevia | 0.021 | 0.032 | 0.069 | 0.021 | 0.021 | 0.000 |
| Pregnancy complication - Miscarriage in a Multiple Pregnancy | 0.004 | 0.005 | 0.025 | 0.004 | 0.004 | 0.000 |
| Pregnancy complication – Other | 0.133 | 0.136 | 0.009 | 0.133 | 0.133 | 0.000 |
| Took folic acid prior to becoming pregnant | 0.698 | 0.583 | 0.240 | 0.698 | 0.697 | 0.000 |
| Took folic acid during first 3 months pregnancy | 0.948 | 0.916 | 0.124 | 0.948 | 0.948 | 0.000 |
| Took iron during pregnancy | 0.695 | 0.711 | 0.036 | 0.695 | 0.695 | 0.000 |
| Number in family smoked during pregnancy | 0.293 | 0.607 | 0.426 | 0.293 | 0.294 | 0.001 |
| Mother currently smokes: Daily | 0.069 | 0.265 | 0.545 | 0.069 | 0.069 | 0.001 |
| Mother currently smokes: Occasionally | 0.050 | 0.077 | 0.111 | 0.050 | 0.050 | 0.000 |
| Mother currently smokes: Not at all | 0.881 | 0.657 | 0.549 | 0.881 | 0.880 | 0.001 |
| Final mode of delivery: Normal delivery | 0.647 | 0.583 | 0.133 | 0.647 | 0.647 | 0.000 |
| Final mode of delivery: Suction assisted birth | 0.104 | 0.088 | 0.056 | 0.104 | 0.104 | 0.000 |
| Final mode of delivery: Forceps assisted birth | 0.038 | 0.042 | 0.020 | 0.038 | 0.038 | 0.000 |
| Final mode of delivery: Planned/Elective caesarean | 0.099 | 0.151 | 0.158 | 0.099 | 0.099 | 0.000 |
| Final mode of delivery: Emergency caesarean | 0.110 | 0.133 | 0.072 | 0.110 | 0.110 | 0.000 |
| Final mode of delivery: Vaginal breech delivery | 0.002 | 0.004 | 0.023 | 0.002 | 0.002 | 0.000 |
| After how many weeks was baby born | 39.689 | 39.444 | 0.124 | 39.689 | 39.689 | 0.000 |
| Baby's weight at birth | 3,551 | 3,458 | 0.177 | 3,551 | 3,550 | 0.000 |
| Birth Complication - No complications during birth | 0.628 | 0.647 | 0.040 | 0.628 | 0.628 | 0.000 |
| Birth Complication - Very Long Labour 12hrs | 0.142 | 0.126 | 0.046 | 0.142 | 0.142 | 0.000 |
| Birth Complication - Very Rapid Labour 2hrs | 0.071 | 0.067 | 0.019 | 0.071 | 0.071 | 0.000 |
| Birth Complication - Foetal Distress Abnormal HR | 0.091 | 0.105 | 0.047 | 0.091 | 0.091 | 0.000 |
| Birth Complication - Foetal Distress Meconium | 0.056 | 0.041 | 0.069 | 0.056 | 0.056 | 0.000 |
| Birth Complication - Foetal Blood Sample During Labour | 0.023 | 0.018 | 0.034 | 0.023 | 0.023 | 0.000 |
| Birth Complication - Birth Injury | 0.016 | 0.014 | 0.016 | 0.016 | 0.016 | 0.000 |
| Birth Complication - Other | 0.100 | 0.099 | 0.005 | 0.100 | 0.100 | 0.000 |
| Household have an illness which affects the baby? | 0.009 | 0.016 | 0.056 | 0.009 | 0.010 | 0.000 |
| Household equivalent annual income | 54,633 | 44,734 | 0.326 | 54,633 | 54,623 | 0.000 |
| Highest Education of Mother - No formal education | 0.002 | 0.004 | 0.037 | 0.002 | 0.002 | 0.000 |
| Highest Education of Mother - Primary education | 0.009 | 0.038 | 0.195 | 0.009 | 0.009 | 0.000 |
| Highest Education of Mother - Lower secondary | 0.027 | 0.166 | 0.486 | 0.027 | 0.027 | 0.002 |
| Highest Education of Mother - Upper secondary | 0.131 | 0.257 | 0.323 | 0.131 | 0.131 | 0.000 |
| Highest Education of Mother - Technical or vocational qualification | 0.052 | 0.107 | 0.203 | 0.052 | 0.052 | 0.000 |
| Highest Education of Mother - Both upper secondary and technical/vocational qualification | 0.037 | 0.049 | 0.062 | 0.037 | 0.037 | 0.000 |
| Highest Education of Mother – Non-degree | 0.186 | 0.187 | 0.003 | 0.186 | 0.186 | 0.000 |
| Highest Education of Mother - Primary Degree | 0.185 | 0.078 | 0.320 | 0.185 | 0.185 | 0.000 |
| Highest Education of Mother - Professional qualification (of Degree status at least) | 0.054 | 0.027 | 0.138 | 0.054 | 0.054 | 0.000 |
| Highest Education of Mother - Both a Degree and a Professional qualification | 0.073 | 0.026 | 0.221 | 0.073 | 0.073 | 0.000 |
| Highest Education of Mother - Postgraduate Certificate or Diploma | 0.091 | 0.033 | 0.241 | 0.091 | 0.091 | 0.000 |
| Highest Education of Mother - Postgraduate Degree (Masters) | 0.137 | 0.025 | 0.420 | 0.137 | 0.137 | 0.000 |
| Highest Education of Mother – Doctorate | 0.017 | 0.004 | 0.129 | 0.017 | 0.017 | 0.000 |
| Hours of sleep during the day | 2.514 | 2.307 | 0.214 | 2.514 | 2.514 | 0.000 |
| Hours of sleep during the night | 10.486 | 10.617 | 0.092 | 10.486 | 10.486 | 0.000 |
| Received their six-week check-up | 0.986 | 0.995 | 0.103 | 0.986 | 0.986 | 0.000 |
| Received vaccines at two months | 0.976 | 0.988 | 0.094 | 0.976 | 0.976 | 0.000 |
| Received vaccines at four months | 0.968 | 0.975 | 0.042 | 0.968 | 0.968 | 0.000 |
| Received vaccines at six months | 0.917 | 0.902 | 0.051 | 0.917 | 0.917 | 0.000 |
| Unvaccinated | 0.009 | 0.002 | 0.086 | 0.009 | 0.009 | 0.000 |

**EBF90days = subsample of infants that were exclusively breastfed for at least 90 days;**

**Non-BF = subsample of infants that were non-breastfed;**

**weighted = weighted by entropy balancing weights to make groups comparable in terms of characteristics.**

**Std. diff. = standardised difference**

**Table A3. Imbalance in covariates by whether breastfed or not breastfed (SA1)**

|  | **BF**  **Mean** | **Non-BF Mean** | **Std. diff.** | **BF Mean (weighted)** | **Non-BF Mean (weighted)** | **Std. diff.** |
| --- | --- | --- | --- | --- | --- | --- |
| Health at birth: Very healthy, no problems | 0.806 | 0.807 | 0.003 | 0.806 | 0.806 | 0.000 |
| Health at birth: Healthy, but a few minor problems | 0.164 | 0.163 | 0.003 | 0.164 | 0.164 | 0.000 |
| Health at birth: Sometimes quite ill | 0.019 | 0.018 | 0.006 | 0.019 | 0.019 | 0.000 |
| Health at birth: Almost always unwell | 0.011 | 0.012 | 0.008 | 0.011 | 0.011 | 0.000 |
| Antenatal care: Shared care (between GP and other professional) | 0.783 | 0.789 | 0.015 | 0.783 | 0.783 | 0.000 |
| Antenatal care: Private consultant alone | 0.136 | 0.104 | 0.099 | 0.136 | 0.136 | 0.000 |
| Antenatal care: Hospital clinic alone | 0.054 | 0.084 | 0.118 | 0.054 | 0.054 | 0.000 |
| Antenatal care: Midwives clinic alone | 0.021 | 0.018 | 0.023 | 0.021 | 0.021 | 0.000 |
| Antenatal care: Independent midwife alone | 0.004 | 0.002 | 0.051 | 0.004 | 0.004 | 0.000 |
| Antenatal care: Had no antenatal care | 0.002 | 0.004 | 0.040 | 0.002 | 0.002 | 0.000 |
| Pregnancy complication - Raised BP in isolation | 0.104 | 0.114 | 0.031 | 0.104 | 0.104 | 0.000 |
| Pregnancy complication - Pre-eclampsia | 0.059 | 0.088 | 0.110 | 0.059 | 0.059 | 0.000 |
| Pregnancy complication - Urinary/Kidney Infection | 0.122 | 0.181 | 0.166 | 0.122 | 0.122 | 0.000 |
| Pregnancy complication - Persistent Vomit/Nausea | 0.163 | 0.194 | 0.079 | 0.163 | 0.163 | 0.000 |
| Pregnancy complication - Gestational Diabetes, diet treated | 0.023 | 0.020 | 0.016 | 0.023 | 0.023 | 0.000 |
| Pregnancy complication - Gestational Diabetes, insulin treated | 0.008 | 0.011 | 0.031 | 0.008 | 0.008 | 0.000 |
| Pregnancy complication - Bleeding during Second Half Pregnancy | 0.054 | 0.066 | 0.052 | 0.054 | 0.054 | 0.000 |
| Pregnancy complication - Vaginal Infection during Pregnancy | 0.038 | 0.032 | 0.033 | 0.038 | 0.038 | 0.000 |
| Pregnancy complication - Intrauterine Growth Restriction (Small baby on scan) | 0.020 | 0.025 | 0.034 | 0.020 | 0.020 | 0.000 |
| Pregnancy complication - Rhesus Incompatibility | 0.039 | 0.038 | 0.008 | 0.039 | 0.039 | 0.000 |
| Pregnancy complication - Influenza | 0.035 | 0.040 | 0.026 | 0.035 | 0.035 | 0.000 |
| Pregnancy complication - Placenta Praevia | 0.024 | 0.032 | 0.045 | 0.024 | 0.024 | 0.000 |
| Pregnancy complication - Miscarriage in a Multiple Pregnancy | 0.004 | 0.005 | 0.020 | 0.004 | 0.004 | 0.000 |
| Pregnancy complication – Other | 0.137 | 0.136 | 0.001 | 0.137 | 0.137 | 0.000 |
| Took folic acid prior to becoming pregnant | 0.667 | 0.583 | 0.174 | 0.667 | 0.667 | 0.000 |
| Took folic acid during first 3 months pregnancy | 0.945 | 0.916 | 0.111 | 0.945 | 0.945 | 0.000 |
| Took iron during pregnancy | 0.708 | 0.711 | 0.008 | 0.708 | 0.708 | 0.000 |
| Number in family smoked during pregnancy | 0.335 | 0.607 | 0.364 | 0.335 | 0.335 | 0.000 |
| Mother currently smokes: Daily | 0.102 | 0.265 | 0.430 | 0.102 | 0.102 | 0.000 |
| Mother currently smokes: Occasionally | 0.068 | 0.077 | 0.034 | 0.068 | 0.068 | 0.000 |
| Mother currently smokes: Not at all | 0.829 | 0.657 | 0.401 | 0.829 | 0.829 | 0.000 |
| Final mode of delivery: Normal delivery | 0.597 | 0.583 | 0.030 | 0.597 | 0.597 | 0.000 |
| Final mode of delivery: Suction assisted birth | 0.107 | 0.088 | 0.066 | 0.107 | 0.107 | 0.000 |
| Final mode of delivery: Forceps assisted birth | 0.049 | 0.042 | 0.034 | 0.049 | 0.049 | 0.000 |
| Final mode of delivery: Planned/Elective caesarean | 0.110 | 0.151 | 0.120 | 0.110 | 0.110 | 0.000 |
| Final mode of delivery: Emergency caesarean | 0.132 | 0.133 | 0.004 | 0.132 | 0.132 | 0.000 |
| Final mode of delivery: Vaginal breech delivery | 0.004 | 0.004 | 0.006 | 0.004 | 0.004 | 0.000 |
| After how many weeks was baby born | 39.530 | 39.444 | 0.041 | 39.530 | 39.530 | 0.000 |
| Baby's weight at birth | 3505 | 3458 | 0.089 | 3505 | 3505 | 0.000 |
| Birth Complication - No complications during birth | 0.605 | 0.647 | 0.087 | 0.605 | 0.605 | 0.000 |
| Birth Complication - Very Long Labour 12hrs | 0.152 | 0.126 | 0.073 | 0.152 | 0.152 | 0.000 |
| Birth Complication - Very Rapid Labour 2hrs | 0.065 | 0.067 | 0.008 | 0.065 | 0.065 | 0.000 |
| Birth Complication - Foetal Distress Abnormal HR | 0.110 | 0.105 | 0.014 | 0.110 | 0.110 | 0.000 |
| Birth Complication - Foetal Distress Meconium | 0.054 | 0.041 | 0.060 | 0.054 | 0.054 | 0.000 |
| Birth Complication - Foetal Blood Sample During Labour | 0.021 | 0.018 | 0.022 | 0.021 | 0.021 | 0.000 |
| Birth Complication - Birth Injury | 0.018 | 0.014 | 0.028 | 0.018 | 0.018 | 0.000 |
| Birth Complication - Other | 0.110 | 0.099 | 0.035 | 0.110 | 0.110 | 0.000 |
| Household have an illness which affects the baby? | 0.010 | 0.016 | 0.048 | 0.010 | 0.010 | 0.000 |
| Household equivalent annual income | 53017 | 44734 | 0.282 | 53017 | 53014 | 0.000 |
| Highest Education of Mother - No formal education | 0.002 | 0.004 | 0.032 | 0.002 | 0.002 | 0.000 |
| Highest Education of Mother - Primary education | 0.011 | 0.038 | 0.179 | 0.011 | 0.011 | 0.000 |
| Highest Education of Mother - Lower secondary | 0.040 | 0.166 | 0.424 | 0.040 | 0.040 | 0.000 |
| Highest Education of Mother - Upper secondary | 0.148 | 0.257 | 0.274 | 0.148 | 0.148 | 0.000 |
| Highest Education of Mother - Technical or vocational qualification | 0.073 | 0.107 | 0.116 | 0.073 | 0.073 | 0.000 |
| Highest Education of Mother - Both upper secondary and technical/vocational qualification | 0.041 | 0.049 | 0.041 | 0.041 | 0.041 | 0.000 |
| Highest Education of Mother – Non-degree | 0.200 | 0.187 | 0.034 | 0.200 | 0.200 | 0.000 |
| Highest Education of Mother - Primary Degree | 0.179 | 0.078 | 0.306 | 0.179 | 0.179 | 0.000 |
| Highest Education of Mother - Professional qualification (of Degree status at least) | 0.050 | 0.027 | 0.120 | 0.050 | 0.050 | 0.000 |
| Highest Education of Mother - Both a Degree and a Professional qualification | 0.070 | 0.026 | 0.210 | 0.070 | 0.070 | 0.000 |
| Highest Education of Mother - Postgraduate Certificate or Diploma | 0.077 | 0.033 | 0.192 | 0.077 | 0.077 | 0.000 |
| Highest Education of Mother - Postgraduate Degree (Masters) | 0.097 | 0.025 | 0.304 | 0.097 | 0.097 | 0.000 |
| Highest Education of Mother – Doctorate | 0.012 | 0.004 | 0.089 | 0.012 | 0.012 | 0.000 |
| Hours of sleep during the day | 2.509 | 2.307 | 0.208 | 2.509 | 2.509 | 0.000 |
| Hours of sleep during the night | 10.532 | 10.617 | 0.060 | 10.532 | 10.532 | 0.000 |
| Received their six-week check-up | 0.991 | 0.995 | 0.056 | 0.991 | 0.991 | 0.000 |
| Received vaccines at two months | 0.985 | 0.988 | 0.027 | 0.985 | 0.985 | 0.000 |
| Received vaccines at four months | 0.979 | 0.975 | 0.029 | 0.979 | 0.979 | 0.000 |
| Received vaccines at six months | 0.929 | 0.902 | 0.095 | 0.929 | 0.929 | 0.000 |
| Unvaccinated | 0.004 | 0.002 | 0.035 | 0.004 | 0.004 | 0.000 |

**EBF90days = subsample of infants that were breastfed;**

**Non-BF = subsample of infants that were non-breastfed;**

**weighted = weighted by entropy balancing weights to make groups comparable in terms of characteristics.**

**Std. diff. = standardised difference**

**Table A4. Imbalance in covariates by whether exclusively breastfed or not exclusively breastfed (SA2)**

|  | **EBF**  **Mean** | **Non-EBF Mean** | **Std. diff.** | **EBF Mean (weighted)** | **Non-EBF Mean (weighted)** | **Std. diff.** |
| --- | --- | --- | --- | --- | --- | --- |
| Health at birth: Very healthy, no problems | 0.823 | 0.792 | 0.081 | 0.823 | 0.823 | 0.000 |
| Health at birth: Healthy, but a few minor problems | 0.151 | 0.175 | 0.066 | 0.151 | 0.151 | 0.000 |
| Health at birth: Sometimes quite ill | 0.017 | 0.020 | 0.020 | 0.017 | 0.017 | 0.000 |
| Health at birth: Almost always unwell | 0.009 | 0.013 | 0.043 | 0.009 | 0.009 | 0.000 |
| Antenatal care: Shared care (between GP and other professional) | 0.778 | 0.792 | 0.034 | 0.778 | 0.778 | 0.000 |
| Antenatal care: Private consultant alone | 0.139 | 0.109 | 0.092 | 0.139 | 0.139 | 0.000 |
| Antenatal care: Hospital clinic alone | 0.053 | 0.078 | 0.100 | 0.053 | 0.053 | 0.000 |
| Antenatal care: Midwives clinic alone | 0.023 | 0.017 | 0.046 | 0.023 | 0.023 | 0.000 |
| Antenatal care: Independent midwife alone | 0.005 | 0.001 | 0.073 | 0.005 | 0.005 | 0.000 |
| Antenatal care: Had no antenatal care | 0.001 | 0.004 | 0.048 | 0.001 | 0.001 | 0.000 |
| Pregnancy complication - Raised BP in isolation | 0.099 | 0.117 | 0.060 | 0.099 | 0.099 | 0.000 |
| Pregnancy complication - Pre-eclampsia | 0.049 | 0.091 | 0.162 | 0.049 | 0.049 | 0.000 |
| Pregnancy complication - Urinary/Kidney Infection | 0.113 | 0.176 | 0.180 | 0.113 | 0.113 | 0.000 |
| Pregnancy complication - Persistent Vomit/Nausea | 0.159 | 0.191 | 0.084 | 0.159 | 0.159 | 0.000 |
| Pregnancy complication - Gestational Diabetes, diet treated | 0.020 | 0.023 | 0.017 | 0.020 | 0.020 | 0.000 |
| Pregnancy complication - Gestational Diabetes, insulin treated | 0.007 | 0.011 | 0.041 | 0.007 | 0.007 | 0.000 |
| Pregnancy complication - Bleeding during Second Half Pregnancy | 0.053 | 0.065 | 0.051 | 0.053 | 0.053 | 0.000 |
| Pregnancy complication - Vaginal Infection during Pregnancy | 0.036 | 0.034 | 0.011 | 0.036 | 0.036 | 0.000 |
| Pregnancy complication - Intrauterine Growth Restriction (Small baby on scan) | 0.015 | 0.028 | 0.086 | 0.015 | 0.015 | 0.000 |
| Pregnancy complication - Rhesus Incompatibility | 0.040 | 0.038 | 0.011 | 0.040 | 0.040 | 0.000 |
| Pregnancy complication - Influenza | 0.036 | 0.037 | 0.006 | 0.036 | 0.036 | 0.000 |
| Pregnancy complication - Placenta Praevia | 0.024 | 0.031 | 0.043 | 0.024 | 0.024 | 0.000 |
| Pregnancy complication - Miscarriage in a Multiple Pregnancy | 0.004 | 0.005 | 0.025 | 0.004 | 0.004 | 0.000 |
| Pregnancy complication - Other | 0.138 | 0.135 | 0.008 | 0.138 | 0.138 | 0.000 |
| Took folic acid prior to becoming pregnant | 0.672 | 0.598 | 0.156 | 0.672 | 0.672 | 0.000 |
| Took folic acid during first 3 months pregnancy | 0.946 | 0.922 | 0.099 | 0.946 | 0.946 | 0.000 |
| Took iron during pregnancy | 0.701 | 0.717 | 0.036 | 0.701 | 0.701 | 0.000 |
| Number in family smoked during pregnancy | 0.326 | 0.552 | 0.310 | 0.326 | 0.326 | 0.000 |
| Mother currently smokes: Daily | 0.100 | 0.230 | 0.356 | 0.100 | 0.100 | 0.000 |
| Mother currently smokes: Occasionally | 0.064 | 0.079 | 0.056 | 0.064 | 0.064 | 0.000 |
| Mother currently smokes: Not at all | 0.835 | 0.691 | 0.345 | 0.835 | 0.835 | 0.000 |
| Final mode of delivery: Normal delivery | 0.615 | 0.570 | 0.091 | 0.615 | 0.615 | 0.000 |
| Final mode of delivery: Suction assisted birth | 0.109 | 0.090 | 0.063 | 0.109 | 0.109 | 0.000 |
| Final mode of delivery: Forceps assisted birth | 0.046 | 0.046 | 0.003 | 0.046 | 0.046 | 0.000 |
| Final mode of delivery: Planned/Elective caesarean | 0.106 | 0.145 | 0.118 | 0.106 | 0.106 | 0.000 |
| Final mode of delivery: Emergency caesarean | 0.120 | 0.144 | 0.069 | 0.120 | 0.120 | 0.000 |
| Final mode of delivery: Vaginal breech delivery | 0.003 | 0.004 | 0.024 | 0.003 | 0.003 | 0.000 |
| After how many weeks was baby born | 39.639 | 39.364 | 0.133 | 39.639 | 39.639 | 0.000 |
| Baby's weight at birth | 3533 | 3443 | 0.168 | 3533 | 3533 | 0.000 |
| Birth Complication - No complications during birth | 0.612 | 0.632 | 0.042 | 0.612 | 0.612 | 0.000 |
| Birth Complication - Very Long Labour 12hrs | 0.147 | 0.136 | 0.031 | 0.147 | 0.147 | 0.000 |
| Birth Complication - Very Rapid Labour 2hrs | 0.067 | 0.064 | 0.013 | 0.067 | 0.067 | 0.000 |
| Birth Complication - Foetal Distress Abnormal HR | 0.105 | 0.110 | 0.016 | 0.105 | 0.105 | 0.000 |
| Birth Complication - Foetal Distress Meconium | 0.054 | 0.043 | 0.052 | 0.054 | 0.054 | 0.000 |
| Birth Complication - Foetal Blood Sample During Labour | 0.019 | 0.020 | 0.011 | 0.019 | 0.019 | 0.000 |
| Birth Complication - Birth Injury | 0.018 | 0.015 | 0.018 | 0.018 | 0.018 | 0.000 |
| Birth Complication - Other | 0.108 | 0.102 | 0.019 | 0.108 | 0.108 | 0.000 |
| Household have an illness which affects the baby? | 0.009 | 0.016 | 0.063 | 0.009 | 0.009 | 0.000 |
| Household equivalent annual income | 53972 | 45748 | 0.273 | 53972 | 53966 | 0.000 |
| Highest Education of Mother - No formal education | 0.001 | 0.004 | 0.054 | 0.001 | 0.001 | 0.000 |
| Highest Education of Mother - Primary education | 0.009 | 0.033 | 0.164 | 0.009 | 0.009 | 0.000 |
| Highest Education of Mother - Lower secondary | 0.035 | 0.142 | 0.387 | 0.035 | 0.035 | 0.001 |
| Highest Education of Mother - Upper secondary | 0.145 | 0.235 | 0.231 | 0.145 | 0.145 | 0.000 |
| Highest Education of Mother - Technical or vocational qualification | 0.073 | 0.100 | 0.098 | 0.073 | 0.073 | 0.000 |
| Highest Education of Mother - Both upper secondary and technical/vocational qualification | 0.038 | 0.050 | 0.058 | 0.038 | 0.038 | 0.000 |
| Highest Education of Mother – Non-degree | 0.201 | 0.189 | 0.029 | 0.201 | 0.201 | 0.000 |
| Highest Education of Mother - Primary Degree | 0.178 | 0.102 | 0.221 | 0.178 | 0.178 | 0.000 |
| Highest Education of Mother - Professional qualification (of Degree status at least) | 0.051 | 0.032 | 0.097 | 0.051 | 0.051 | 0.000 |
| Highest Education of Mother - Both a Degree and a Professional qualification | 0.072 | 0.034 | 0.166 | 0.072 | 0.072 | 0.000 |
| Highest Education of Mother - Postgraduate Certificate or Diploma | 0.081 | 0.039 | 0.177 | 0.081 | 0.081 | 0.000 |
| Highest Education of Mother - Postgraduate Degree (Masters) | 0.105 | 0.035 | 0.277 | 0.105 | 0.105 | 0.000 |
| Highest Education of Mother - Doctorate | 0.012 | 0.005 | 0.077 | 0.012 | 0.012 | 0.000 |
| Hours of sleep during the day | 2.503 | 2.359 | 0.149 | 2.503 | 2.503 | 0.000 |
| Hours of sleep during the night | 10.558 | 10.574 | 0.011 | 10.558 | 10.558 | 0.000 |
| Received their six-week check-up | 0.990 | 0.995 | 0.055 | 0.990 | 0.990 | 0.000 |
| Received vaccines at two months | 0.984 | 0.989 | 0.046 | 0.984 | 0.984 | 0.000 |
| Received vaccines at four months | 0.978 | 0.977 | 0.003 | 0.978 | 0.978 | 0.000 |
| Received vaccines at six months | 0.927 | 0.909 | 0.066 | 0.927 | 0.927 | 0.000 |
| Unvaccinated | 0.005 | 0.002 | 0.040 | 0.005 | 0.005 | 0.000 |

**EBF = subsample of infants that were exclusively breastfed;**

**Non-EBF = subsample of infants that were non-exclusively breastfed;**

**weighted = weighted by entropy balancing weights to make groups comparable in terms of characteristics.**

**Std. diff. = standardised difference**

**Table A5. Imbalance in covariates by whether exclusively breastfed for at least 90 days or not exclusively breastfed (SA3)**

|  | **EBF90days Mean** | **Non-EBF Mean** | **Std. diff.** | **EBF90days Mean (weighted)** | **Non-EBF Mean (weighted)** | **Std. diff.** |
| --- | --- | --- | --- | --- | --- | --- |
| Health at birth: Very healthy, no problems | 0.844 | 0.796 | 0.127 | 0.844 | 0.844 | 0.000 |
| Health at birth: Healthy, but a few minor problems | 0.133 | 0.172 | 0.109 | 0.133 | 0.133 | 0.000 |
| Health at birth: Sometimes quite ill | 0.014 | 0.020 | 0.043 | 0.014 | 0.014 | 0.000 |
| Health at birth: Almost always unwell | 0.008 | 0.012 | 0.037 | 0.008 | 0.008 | 0.000 |
| Antenatal care: Shared care (between GP and other professional) | 0.782 | 0.786 | 0.009 | 0.782 | 0.782 | 0.000 |
| Antenatal care: Private consultant alone | 0.134 | 0.120 | 0.041 | 0.134 | 0.134 | 0.000 |
| Antenatal care: Hospital clinic alone | 0.048 | 0.072 | 0.098 | 0.048 | 0.048 | 0.000 |
| Antenatal care: Midwives clinic alone | 0.027 | 0.018 | 0.061 | 0.027 | 0.027 | 0.000 |
| Antenatal care: Independent midwife alone | 0.008 | 0.002 | 0.092 | 0.008 | 0.008 | 0.000 |
| Antenatal care: Had no antenatal care | 0.001 | 0.003 | 0.047 | 0.001 | 0.001 | 0.000 |
| Pregnancy complication - Raised BP in isolation | 0.089 | 0.114 | 0.082 | 0.089 | 0.089 | 0.000 |
| Pregnancy complication - Pre-eclampsia | 0.040 | 0.080 | 0.167 | 0.040 | 0.040 | 0.000 |
| Pregnancy complication - Urinary/Kidney Infection | 0.097 | 0.160 | 0.189 | 0.097 | 0.097 | 0.000 |
| Pregnancy complication - Persistent Vomit/Nausea | 0.154 | 0.182 | 0.075 | 0.154 | 0.154 | 0.000 |
| Pregnancy complication - Gestational Diabetes, diet treated | 0.021 | 0.022 | 0.004 | 0.021 | 0.021 | 0.000 |
| Pregnancy complication - Gestational Diabetes, insulin treated | 0.008 | 0.009 | 0.019 | 0.008 | 0.008 | 0.000 |
| Pregnancy complication - Bleeding during Second Half Pregnancy | 0.046 | 0.063 | 0.077 | 0.046 | 0.046 | 0.000 |
| Pregnancy complication - Vaginal Infection during Pregnancy | 0.036 | 0.035 | 0.005 | 0.036 | 0.036 | 0.000 |
| Pregnancy complication - Intrauterine Growth Restriction (Small baby on scan) | 0.014 | 0.024 | 0.076 | 0.014 | 0.014 | 0.000 |
| Pregnancy complication - Rhesus Incompatibility | 0.034 | 0.040 | 0.029 | 0.034 | 0.034 | 0.000 |
| Pregnancy complication - Influenza | 0.037 | 0.037 | 0.003 | 0.037 | 0.037 | 0.000 |
| Pregnancy complication - Placenta Praevia | 0.021 | 0.029 | 0.055 | 0.021 | 0.021 | 0.000 |
| Pregnancy complication - Miscarriage in a Multiple Pregnancy | 0.004 | 0.005 | 0.017 | 0.004 | 0.004 | 0.000 |
| Pregnancy complication - Other | 0.133 | 0.138 | 0.012 | 0.133 | 0.133 | 0.000 |
| Took folic acid prior to becoming pregnant | 0.698 | 0.615 | 0.175 | 0.698 | 0.697 | 0.000 |
| Took folic acid during first 3 months pregnancy | 0.948 | 0.929 | 0.076 | 0.948 | 0.947 | 0.000 |
| Took iron during pregnancy | 0.695 | 0.713 | 0.040 | 0.695 | 0.695 | 0.000 |
| Number in family smoked during pregnancy | 0.293 | 0.488 | 0.280 | 0.293 | 0.294 | 0.001 |
| Mother currently smokes: Daily | 0.069 | 0.197 | 0.382 | 0.069 | 0.069 | 0.001 |
| Mother currently smokes: Occasionally | 0.050 | 0.078 | 0.115 | 0.050 | 0.050 | 0.000 |
| Mother currently smokes: Not at all | 0.881 | 0.725 | 0.399 | 0.881 | 0.880 | 0.001 |
| Final mode of delivery: Normal delivery | 0.647 | 0.575 | 0.148 | 0.647 | 0.647 | 0.000 |
| Final mode of delivery: Suction assisted birth | 0.104 | 0.098 | 0.020 | 0.104 | 0.104 | 0.000 |
| Final mode of delivery: Forceps assisted birth | 0.038 | 0.049 | 0.052 | 0.038 | 0.038 | 0.000 |
| Final mode of delivery: Planned/Elective caesarean | 0.099 | 0.135 | 0.113 | 0.099 | 0.099 | 0.000 |
| Final mode of delivery: Emergency caesarean | 0.110 | 0.139 | 0.089 | 0.110 | 0.110 | 0.000 |
| Final mode of delivery: Vaginal breech delivery | 0.002 | 0.004 | 0.034 | 0.002 | 0.002 | 0.000 |
| After how many weeks was baby born | 39.689 | 39.439 | 0.124 | 39.689 | 39.689 | 0.000 |
| Baby's weight at birth | 3551 | 3467 | 0.157 | 3551 | 3550 | 0.000 |
| Birth Complication - No complications during birth | 0.628 | 0.621 | 0.015 | 0.628 | 0.628 | 0.000 |
| Birth Complication - Very Long Labour 12hrs | 0.142 | 0.141 | 0.002 | 0.142 | 0.142 | 0.000 |
| Birth Complication - Very Rapid Labour 2hrs | 0.071 | 0.064 | 0.030 | 0.071 | 0.071 | 0.000 |
| Birth Complication - Foetal Distress Abnormal HR | 0.091 | 0.113 | 0.071 | 0.091 | 0.091 | 0.000 |
| Birth Complication - Foetal Distress Meconium | 0.056 | 0.046 | 0.042 | 0.056 | 0.056 | 0.000 |
| Birth Complication - Foetal Blood Sample During Labour | 0.023 | 0.019 | 0.027 | 0.023 | 0.023 | 0.000 |
| Birth Complication - Birth Injury | 0.016 | 0.016 | 0.001 | 0.016 | 0.016 | 0.000 |
| Birth Complication - Other | 0.100 | 0.107 | 0.021 | 0.100 | 0.100 | 0.000 |
| Household have an illness which affects the baby? | 0.009 | 0.013 | 0.037 | 0.009 | 0.010 | 0.000 |
| Household equivalent annual income | 54633 | 48243 | 0.203 | 54633 | 54618 | 0.000 |
| Highest Education of Mother - No formal education | 0.002 | 0.003 | 0.024 | 0.002 | 0.002 | 0.000 |
| Highest Education of Mother - Primary education | 0.009 | 0.025 | 0.130 | 0.009 | 0.009 | 0.000 |
| Highest Education of Mother - Lower secondary | 0.027 | 0.109 | 0.333 | 0.027 | 0.027 | 0.003 |
| Highest Education of Mother - Upper secondary | 0.131 | 0.209 | 0.211 | 0.131 | 0.131 | 0.000 |
| Highest Education of Mother - Technical or vocational qualification | 0.052 | 0.097 | 0.172 | 0.052 | 0.052 | 0.000 |
| Highest Education of Mother - Both upper secondary and technical/vocational qualification | 0.037 | 0.046 | 0.049 | 0.037 | 0.037 | 0.000 |
| Highest Education of Mother – Non-degree | 0.186 | 0.197 | 0.029 | 0.186 | 0.186 | 0.000 |
| Highest Education of Mother - Primary Degree | 0.185 | 0.125 | 0.166 | 0.185 | 0.185 | 0.000 |
| Highest Education of Mother - Professional qualification (of Degree status at least) | 0.054 | 0.037 | 0.083 | 0.054 | 0.054 | 0.000 |
| Highest Education of Mother - Both a Degree and a Professional qualification | 0.073 | 0.046 | 0.114 | 0.073 | 0.073 | 0.000 |
| Highest Education of Mother - Postgraduate Certificate or Diploma | 0.091 | 0.050 | 0.161 | 0.091 | 0.091 | 0.000 |
| Highest Education of Mother - Postgraduate Degree (Masters) | 0.137 | 0.048 | 0.312 | 0.137 | 0.137 | 0.000 |
| Highest Education of Mother - Doctorate | 0.017 | 0.006 | 0.101 | 0.017 | 0.017 | 0.000 |
| Hours of sleep during the day | 2.514 | 2.403 | 0.116 | 2.514 | 2.514 | 0.000 |
| Hours of sleep during the night | 10.486 | 10.589 | 0.073 | 10.486 | 10.486 | 0.000 |
| Received their six-week check-up | 0.986 | 0.995 | 0.094 | 0.986 | 0.986 | 0.000 |
| Received vaccines at two months | 0.976 | 0.990 | 0.105 | 0.976 | 0.976 | 0.000 |
| Received vaccines at four months | 0.968 | 0.980 | 0.078 | 0.968 | 0.968 | 0.000 |
| Received vaccines at six months | 0.917 | 0.918 | 0.006 | 0.917 | 0.917 | 0.000 |
| Unvaccinated | 0.009 | 0.002 | 0.092 | 0.009 | 0.009 | 0.000 |

**EBF90days = subsample of infants that were exclusively breastfed for at least 90 days;**

**Non-EBF = subsample of infants that were non- exclusively breastfed;**

**weighted = weighted by entropy balancing weights to make groups comparable in terms of characteristics.**

**Std. diff. = standardised difference**

**Table A6. Difference in outcomes by group after weighting to ensure covariate balance using Entropy Balancing weights**

|  | **BF**  **versus**  **non-BF (SA1) Difference** | **95% Confidence interval** | **EBF versus Non-EBF (SA2) Difference** | **95% Confidence interval** | **EBF90days versus**  **Non-EBF (SA3) Difference** | **95% Confidence interval** |
| --- | --- | --- | --- | --- | --- | --- |
| Average number of nights spent in hospital by baby | -0.142 | (-0.267, -0.016) | -0.168 | (-0.283, -0.053) | -0.252 | (-0.379, -0.124) |
| Current health of the baby | -0.009 | (-0.026, 0.007) | -0.015 | (-0.031, 0.002) | -0.022 | (-0.041, -0.003) |
| Baby admitted to hospital | -0.027 | (-0.040, -0.014) | -0.023 | (-0.036, -0.010) | -0.038 | (-0.053, -0.024) |
| Respiratory disease [including asthma] | -0.018 | (-0.025, -0.010) | -0.011 | (-0.018, -0.004) | -0.017 | (-0.025, -0.009) |
| Digestive allergies (e.g. lactose intolerant) | -0.003 | (-0.011, 0.005) | -0.001 | (-0.009, 0.006) | -0.004 | (-0.013, 0.005) |
| Eczema or any kind of skin allergy | -0.018 | (-0.031, -0.005) | -0.025 | (-0.038, -0.012) | -0.031 | (-0.047, -0.016) |
| Kidney disease | 0.000 | (-0.003, 0.003) | 0.001 | (-0.002, 0.004) | -0.001 | (-0.004, 0.003) |
| Any developmental delay | -0.004 | (-0.007, 0.000) | -0.003 | (-0.007, 0.000) | -0.003 | (-0.007, 0.001) |
| Snuffles/common cold | -0.014 | (-0.034, 0.005) | -0.032 | (-0.052, -0.012) | -0.051 | (-0.073, -0.028) |
| Chest infection | -0.059 | (-0.077, -0.040) | -0.045 | (-0.063, -0.027) | -0.087 | (-0.107, -0.066) |
| Ear infection | -0.043 | (-0.057, -0.028) | -0.027 | (-0.042, -0.013) | -0.040 | (-0.057, -0.023) |
| Feeding problems | -0.010 | (-0.021, 0.001) | -0.014 | (-0.024, -0.003) | -0.034 | (-0.046, -0.023) |
| Sleeping problems | -0.007 | (-0.013, 0.000) | -0.008 | (-0.014, -0.002) | -0.013 | (-0.020, -0.006) |
| Dental problems | 0.004 | (-0.003, 0.010) | 0.002 | (-0.004, 0.008) | 0.003 | (-0.005, 0.010) |
| Wheezing or asthma | -0.032 | (-0.043, -0.021) | -0.026 | (-0.037, -0.016) | -0.036 | (-0.048, -0.025) |
| Skin problem | -0.019 | (-0.033, -0.005) | -0.023 | (-0.038, -0.009) | -0.019 | (-0.036, -0.002) |
| Persistent nappy rash | 0.003 | (-0.004, 0.009) | 0.000 | (-0.006, 0.007) | 0.006 | (-0.002, 0.014) |
| Failure to gain weight or to grow | 0.016 | (0.010, 0.022) | 0.013 | (0.007, 0.019) | 0.009 | (0.002, 0.015) |
| Persistent vomiting | -0.004 | (-0.013, 0.005) | -0.006 | (-0.014, 0.003) | -0.015 | (-0.025, -0.005) |
| Persistent diarrhoea/Constipation | 0.004 | (-0.006, 0.013) | -0.005 | (-0.015, 0.005) | -0.009 | (-0.020, 0.002) |
| Meningitis | 0.002 | (0.000, 0.004) | 0.002 | (0.000, 0.004) | 0.000 | (-0.002, 0.002) |
| Colic | -0.007 | (-0.018, 0.003) | -0.009 | (-0.019, 0.001) | -0.024 | (-0.035, -0.013) |
| Number of observations | **9,879** |  | **9,879** |  | **7,375** |  |

**Table A7. Unadjusted difference in outcomes by group**

|  | **EBF90days versus**  **Non-BF Difference** | **95% Confidence interval** | **BF**  **versus**  **non-BF (SA1) Difference** | **95% Confidence interval** | **EBF**  **versus**  **Non-EBF**  **(SA2)**  **Difference** | **95% Confidence interval** | **EBF90days versus**  **Non-EBF**  **(SA3)**  **Difference** | **95% Confidence interval** |
| --- | --- | --- | --- | --- | --- | --- | --- | --- |
| Average number of nights spent in hospital by baby | -0.331 | (-0.486, -0.176) | -0.161 | (-0.287, -0.034) | -0.278 | (-0.402, -0.153) | -0.368 | (-0.530, -0.206) |
| Current health of the baby | -0.020 | (-0.042, 0.001) | -0.004 | (-0.021, 0.013) | -0.021 | (-0.038, -0.004) | -0.028 | (-0.049, -0.007) |
| Baby admitted to hospital | -0.058 | (-0.075, -0.04) | -0.035 | (-0.048, -0.021) | -0.038 | (-0.051, -0.024) | -0.055 | (-0.072, -0.038) |
| Respiratory disease [including asthma] | -0.032 | (-0.042, -0.021) | -0.024 | (-0.032, -0.017) | -0.019 | (-0.027, -0.012) | -0.026 | (-0.036, -0.017) |
| Digestive allergies (e.g. lactose intolerant) | -0.002 | (-0.012, 0.008) | 0.002 | (-0.006, 0.010) | 0.000 | (-0.008, 0.008) | -0.003 | (-0.013, 0.006) |
| Eczema or any kind of skin allergy | -0.016 | (-0.034, 0.001) | -0.009 | (-0.022, 0.005) | -0.017 | (-0.030, -0.004) | -0.019 | (-0.036, -0.003) |
| Kidney disease | 0.001 | (-0.002, 0.005) | 0.002 | (-0.001, 0.005) | 0.002 | (-0.001, 0.005) | 0.001 | (-0.003, 0.004) |
| Any developmental delay | -0.003 | (-0.008, 0.002) | -0.002 | (-0.006, 0.001) | -0.004 | (-0.007, 0.000) | -0.004 | (-0.008, 0.001) |
| Snuffles/common cold | -0.090 | (-0.116, -0.064) | -0.052 | (-0.072, -0.032) | -0.065 | (-0.084, -0.045) | -0.090 | (-0.115, -0.065) |
| Chest infection | -0.128 | (-0.152, -0.104) | -0.080 | (-0.099, -0.062) | -0.069 | (-0.087, -0.051) | -0.113 | (-0.136, -0.090) |
| Ear infection | -0.061 | (-0.081, -0.041) | -0.046 | (-0.061, -0.031) | -0.038 | (-0.053, -0.023) | -0.052 | (-0.071, -0.033) |
| Feeding problems | -0.036 | (-0.049, -0.022) | -0.009 | (-0.020, 0.001) | -0.015 | (-0.026, -0.005) | -0.037 | (-0.051, -0.024) |
| Sleeping problems | -0.012 | (-0.020, -0.003) | -0.007 | (-0.014, -0.001) | -0.009 | (-0.015, -0.002) | -0.012 | (-0.02, -0.003) |
| Dental problems | 0.002 | (-0.007, 0.010) | 0.001 | (-0.006, 0.008) | 0.001 | (-0.006, 0.007) | 0.001 | (-0.007, 0.010) |
| Wheezing or asthma | -0.060 | (-0.075, -0.045) | -0.046 | (-0.057, -0.035) | -0.042 | (-0.053, -0.031) | -0.052 | (-0.067, -0.038) |
| Skin problem | -0.006 | (-0.024, 0.013) | -0.009 | (-0.023, 0.005) | -0.014 | (-0.028, 0.000) | -0.007 | (-0.025, 0.011) |
| Persistent nappy rash | -0.004 | (-0.013, 0.006) | -0.007 | (-0.014, 0.000) | -0.007 | (-0.014, 0.000) | -0.003 | (-0.012, 0.006) |
| Failure to gain weight or to grow | 0.011 | (0.004, 0.018) | 0.015 | (0.009, 0.021) | 0.012 | (0.006, 0.018) | 0.008 | (0.001, 0.015) |
| Persistent vomiting | -0.009 | (-0.020, 0.002) | 0.003 | (-0.006, 0.012) | -0.002 | (-0.011, 0.006) | -0.012 | (-0.023, -0.001) |
| Persistent diarrhoea/Constipation | -0.008 | (-0.021, 0.005) | 0.000 | (-0.010, 0.010) | -0.006 | (-0.016, 0.004) | -0.011 | (-0.023, 0.001) |
| Meningitis | -0.000 | (-0.003, 0.002) | 0.002 | (-0.001, 0.004) | 0.002 | (-0.001, 0.004) | 0.000 | (-0.003, 0.002) |
| Colic | -0.026 | (-0.039, -0.013) | -0.007 | (-0.017, 0.004) | -0.013 | (-0.023, -0.002) | -0.028 | (-0.041, -0.016) |
| Number of observations | **6,199** |  | **9,879** |  | **9,879** |  | **7,375** |  |

**Table A8. Difference in outcomes by group after matching based on propensity score to ensure covariate balance**

|  | **EBF90days versus**  **Non-BF Difference** | **95% Confidence interval** | **BF**  **versus non-BF**  **(SA1) Difference** | **95% Confidence interval** | **EBF**  **versus**  **Non-EBF**  **(SA2) Difference** | **95% Confidence interval** | **EBF90days versus**  **Non-EBF**  **(SA3) Difference** | **95% Confidence interval** |
| --- | --- | --- | --- | --- | --- | --- | --- | --- |
| Average number of nights spent in hospital by baby | -0.373 | (-0.671, -0.075) | -0.150 | (-0.421, 0.120) | -0.094 | (-0.271, 0.082) | -0.256 | (-0.447, -0.065) |
| Current health of the baby | -0.023 | (-0.061, 0.015) | -0.008 | (-0.032, 0.017) | -0.005 | (-0.027, 0.017) | -0.028 | (-0.061, 0.004) |
| Baby admitted to hospital | -0.041 | (-0.068, -0.015) | -0.023 | (-0.045, -0.001) | -0.021 | (-0.040, -0.001) | -0.041 | (-0.066, -0.017) |
| Respiratory disease [including asthma] | -0.028 | (-0.046, -0.010) | -0.020 | (-0.031, -0.009) | -0.009 | (-0.020, 0.001) | -0.014 | (-0.028, 0.000) |
| Digestive allergies (e.g. lactose intolerant) | -0.013 | (-0.029, 0.003) | -0.008 | (-0.023, 0.007) | -0.003 | (-0.014, 0.008) | -0.005 | (-0.022, 0.011) |
| Eczema or any kind of skin allergy | -0.039 | (-0.067, -0.010) | -0.011 | (-0.034, 0.011) | -0.033 | (-0.053, -0.013) | -0.023 | (-0.050, 0.003) |
| Kidney disease | 0.004 | (-0.002, 0.010) | 0.001 | (-0.004, 0.006) | -0.001 | (-0.005, 0.004) | 0.000 | (-0.004, 0.005) |
| Any developmental delay | -0.003 | (-0.010, 0.005) | 0.000 | (-0.006, 0.005) | -0.006 | (-0.011, 0.000) | -0.001 | (-0.007, 0.005) |
| Snuffles/common cold | -0.052 | (-0.094, -0.009) | -0.002 | (-0.035, 0.031) | -0.031 | (-0.060, -0.001) | -0.077 | (-0.115, -0.038) |
| Chest infection | -0.091 | (-0.130, -0.053) | -0.065 | (-0.094, -0.036) | -0.033 | (-0.059, -0.006) | -0.108 | (-0.145, -0.072) |
| Ear infection | -0.050 | (-0.082, -0.019) | -0.043 | (-0.067, -0.019) | -0.017 | (-0.038, 0.004) | -0.033 | (-0.060, -0.005) |
| Feeding problems | -0.038 | (-0.057, -0.018) | -0.019 | (-0.039, 0.000) | -0.006 | (-0.021, 0.009) | -0.035 | (-0.054, -0.016) |
| Sleeping problems | -0.009 | (-0.022, 0.005) | -0.007 | (-0.019, 0.006) | -0.007 | (-0.016, 0.003) | -0.010 | (-0.024, 0.004) |
| Dental problems | 0.003 | (-0.011, 0.016) | 0.002 | (-0.008, 0.012) | 0.000 | (-0.009, 0.010) | 0.009 | (-0.002, 0.019) |
| Wheezing or asthma | -0.044 | (-0.071, -0.017) | -0.029 | (-0.046, -0.012) | -0.021 | (-0.037, -0.006) | -0.031 | (-0.052, -0.010) |
| Skin problem | -0.027 | (-0.057, 0.004) | -0.017 | (-0.042, 0.007) | -0.027 | (-0.048, -0.005) | -0.012 | (-0.041, 0.017) |
| Persistent nappy rash | 0.008 | (-0.006, 0.021) | 0.003 | (-0.007, 0.013) | 0.000 | (-0.009, 0.009) | 0.015 | (0.003, 0.027) |
| Failure to gain weight or to grow | 0.009 | (-0.001, 0.020) | 0.012 | (0.003, 0.022) | 0.013 | (0.005, 0.020) | 0.011 | (0.000, 0.022) |
| Persistent vomiting | -0.023 | (-0.042, -0.004) | -0.010 | (-0.026, 0.007) | -0.004 | (-0.017, 0.008) | -0.014 | (-0.031, 0.003) |
| Persistent diarrhoea/Constipation | -0.011 | (-0.032, 0.010) | -0.002 | (-0.019, 0.015) | -0.010 | (-0.025, 0.006) | 0.000 | (-0.019, 0.018) |
| Meningitis | 0.001 | (-0.002, 0.004) | 0.003 | (0.001, 0.005) | 0.003 | (0.000, 0.005) | 0.001 | (-0.002, 0.003) |
| Colic | -0.029 | (-0.051, -0.008) | -0.009 | (-0.027, 0.009) | -0.006 | (-0.021, 0.010) | -0.015 | (-0.033, 0.003) |
| Number of observations | **6,199** |  | **9,879** |  | **9,879** |  | **7,375** |  |

**Table A9. Descriptive statistics for covariates for the analysis sample as a whole (N=9,879):**

|  | **median [IQR]** |
| --- | --- |
| Household equivalent annual income | 45,600 [62,400; 29,001] |
| After how many weeks was baby born | 40 [41; 39] |
| Baby's weight at birth | 3,500 [3,800; 3,200] |
| Number in family smoked during pregnancy | 0 [1; 0] |
| Household have an illness which affects the baby? | 0 [0; 0] |
| Hours of sleep during the day | 2 [3; 2] |
| Hours of sleep during the night | 11 [12; 10] |
|  | **%** |
| Health at birth: Very healthy, no problems | 80.68% |
| Health at birth: Healthy, but a few minor problems | 16.34% |
| Health at birth: Sometimes quite ill | 1.88% |
| Health at birth: Almost always unwell | 1.10% |
| Antenatal care: Shared care (between GP and other professional) | 78.50% |
| Antenatal care: Private consultant alone | 12.32% |
| Antenatal care: Hospital clinic alone | 6.64% |
| Antenatal care: Midwives clinic alone | 1.97% |
| Antenatal care: Independent midwife alone | 0.31% |
| Antenatal care: Had no antenatal care | 0.25% |
| Pregnancy complication - Raised BP in isolation | 10.83% |
| Pregnancy complication - Pre-eclampsia | 7.10% |
| Pregnancy complication - Urinary/Kidney Infection | 14.57% |
| Pregnancy complication - Persistent Vomit/Nausea | 17.55% |
| Pregnancy complication - Gestational Diabetes, diet treated | 2.17% |
| Pregnancy complication - Gestational Diabetes, insulin treated | 0.90% |
| Pregnancy complication - Bleeding during Second Half Pregnancy | 5.91% |
| Pregnancy complication - Vaginal Infection during Pregnancy | 3.54% |
| Pregnancy complication - Intrauterine Growth Restriction (Small baby on scan) | 2.16% |
| Pregnancy complication - Rhesus Incompatibility | 3.86% |
| Pregnancy complication - Influenza | 3.66% |
| Pregnancy complication - Placenta Praevia | 2.74% |
| Pregnancy complication - Miscarriage in a Multiple Pregnancy | 0.45% |
| Pregnancy complication – Other | 13.67% |
| Took folic acid prior to becoming pregnant | 63.34% |
| Took folic acid during first 3 months pregnancy | 93.33% |
| Took iron during pregnancy | 70.91% |
| Mother currently smokes: Daily | 16.81% |
| Mother currently smokes: Occasionally | 7.20% |
| Mother currently smokes: Not at all | 75.99% |
| Final mode of delivery: Normal delivery | 59.16% |
| Final mode of delivery: Suction assisted birth | 9.92% |
| Final mode of delivery: Forceps assisted birth | 4.62% |
| Final mode of delivery: Planned/Elective caesarean | 12.66% |
| Final mode of delivery: Emergency caesarean | 13.27% |
| Final mode of delivery: Vaginal breech delivery | 0.37% |
| Birth Complication - No complications during birth | 62.22% |
| Birth Complication - Very Long Labour 12hrs | 14.13% |
| Birth Complication - Very Rapid Labour 2hrs | 6.56% |
| Birth Complication - Foetal Distress Abnormal HR | 10.79% |
| Birth Complication - Foetal Distress Meconium | 4.85% |
| Birth Complication - Foetal Blood Sample During Labour | 1.96% |
| Birth Complication - Birth Injury | 1.64% |
| Birth Complication - Other | 10.53% |
| Highest Education of Mother - No formal education | 0.27% |
| Highest Education of Mother - Primary education | 2.16% |
| Highest Education of Mother - Lower secondary | 9.09% |
| Highest Education of Mother - Upper secondary | 19.17% |
| Highest Education of Mother - Technical or vocational qualification | 8.69% |
| Highest Education of Mother - Both upper secondary and technical/vocational qualification | 4.41% |
| Highest Education of Mother – Non-degree | 19.48% |
| Highest Education of Mother - Primary Degree | 13.85% |
| Highest Education of Mother - Professional qualification (of Degree status at least) | 4.08% |
| Highest Education of Mother - Both a Degree and a Professional qualification | 5.22% |
| Highest Education of Mother - Postgraduate Certificate or Diploma | 5.94% |
| Highest Education of Mother - Postgraduate Degree (Masters) | 6.80% |
| Highest Education of Mother – Doctorate | 0.84% |
| Received their six-week check-up | 99.27% |
| Received vaccines at two months | 98.66% |
| Received vaccines at four months | 97.75% |
| Received vaccines at six months | 91.80% |
| Unvaccinated | 0.34% |
|  |  |

**Table A10. Descriptive statistics for outcomes by group**

|  | **All** | **EBF90days** | **EFB** | **BF** |
| --- | --- | --- | --- | --- |
|  | **Mean [IQR]** | **Mean [IQR]** | **Mean [IQR]** | **Mean [IQR]** |
| Average number of nights spent in hospital by baby | 0.62 [0, 0] | 0.39 [0, 0] | 0.48 [0, 0] | 0.56 [0, 0] |
| Current health of the baby | 1.18 [1, 1] | 1.16 [1, 1] | 1.17 [1, 1] | 1.18 [1, 1] |
|  | **%** | **%** | **%** | **%** |
| Baby admitted to hospital | 12.83% | 9.13% | 10.86% | 11.42% |
| Respiratory disease [including asthma] | 3.80% | 2.08% | 2.78% | 2.82% |
| Digestive allergies (e.g. lactose intolerant) | 3.88% | 3.57% | 3.88% | 3.95% |
| Eczema or any kind of skin allergy | 12.70% | 11.57% | 11.81% | 12.36% |
| Kidney disease | 0.56% | 0.54% | 0.66% | 0.64% |
| Any developmental delay | 0.82% | 0.63% | 0.61% | 0.73% |
| Snuffles/common cold | 46.59% | 40.69% | 43.21% | 44.48% |
| Chest infection | 31.62% | 23.64% | 28.01% | 28.38% |
| Ear infection | 17.01% | 13.65% | 15.01% | 15.16% |
| Feeding problems | 7.99% | 4.97% | 7.19% | 7.60% |
| Sleeping problems | 2.77% | 2.03% | 2.31% | 2.48% |
| Dental problems | 2.74% | 2.85% | 2.78% | 2.78% |
| Wheezing or asthma | 8.55% | 5.29% | 6.38% | 6.69% |
| Skin problem | 15.08% | 15.05% | 14.33% | 14.71% |
| Persistent nappy rash | 3.29% | 3.35% | 2.91% | 3.00% |
| Failure to gain weight or to grow | 2.37% | 2.58% | 3.01% | 2.99% |
| Persistent vomiting | 5.25% | 4.16% | 5.13% | 5.38% |
| Persistent diarrhoea/Constipation | 6.56% | 5.74% | 6.23% | 6.57% |
| Meningitis | 0.34% | 0.23% | 0.42% | 0.41% |
| Colic | 7.24% | 5.02% | 6.57% | 6.96% |
| Number of observations | **9,879** | **2,212** | **4,716** | **5,892** |
