# Supplementary material for: Exploring the relationship between breastfeeding and the incidence of infant illnesses in Ireland: evidence from a nationally representative prospective cohort study
Source: BMC Public Health. 2023 Jan 20;23:140. doi: 10.1186/s12889-023-15045-8 (PMC9854149; doi:10.1186/s12889-023-15045-8)
Supplement: Supplementary file 2 — Additional file 2. [file 12889_2023_15045_MOESM2_ESM.docx]

**Figure A1. Entropy Balanced comparison of outcomes for BF versus non-BF (SA1) rescaled by non-BF standard deviation**


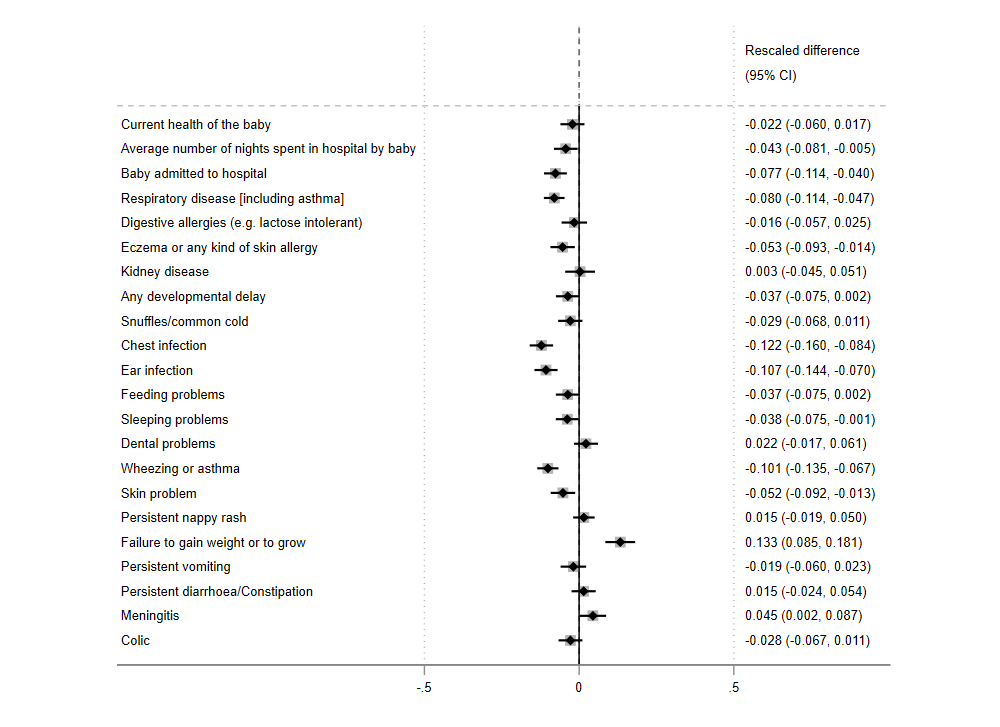


**Figure A2. Entropy Balanced comparison of outcomes for EBF versus non-EBF (SA2) rescaled by non-BF standard deviation**


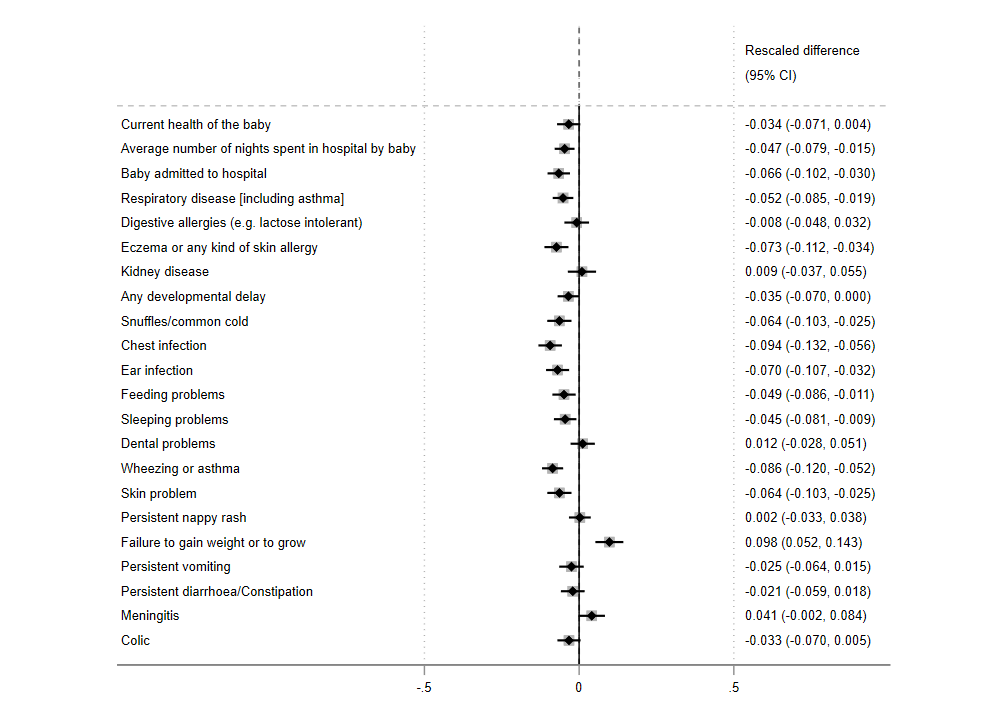


**Figure A3. Entropy Balanced comparison of outcomes for EBF (90+ days) versus non-EBF (SA3) rescaled by non-BF standard deviation**


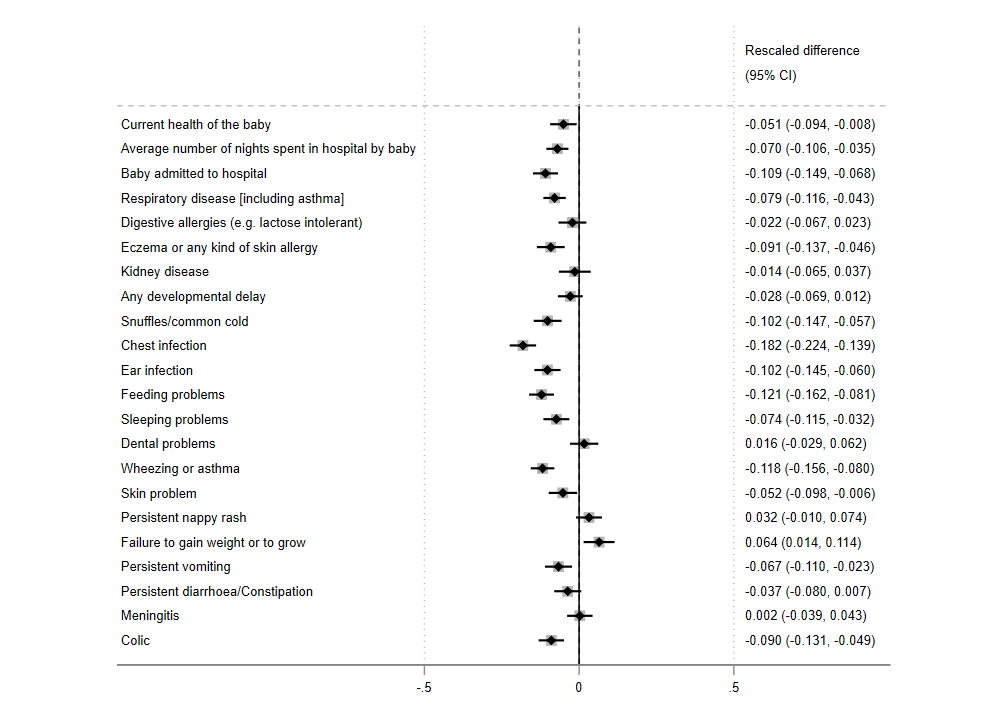


**Figure A4. Unadjusted comparison of outcomes for EBF (90+ days) versus non-BF rescaled by non-BF standard deviation**


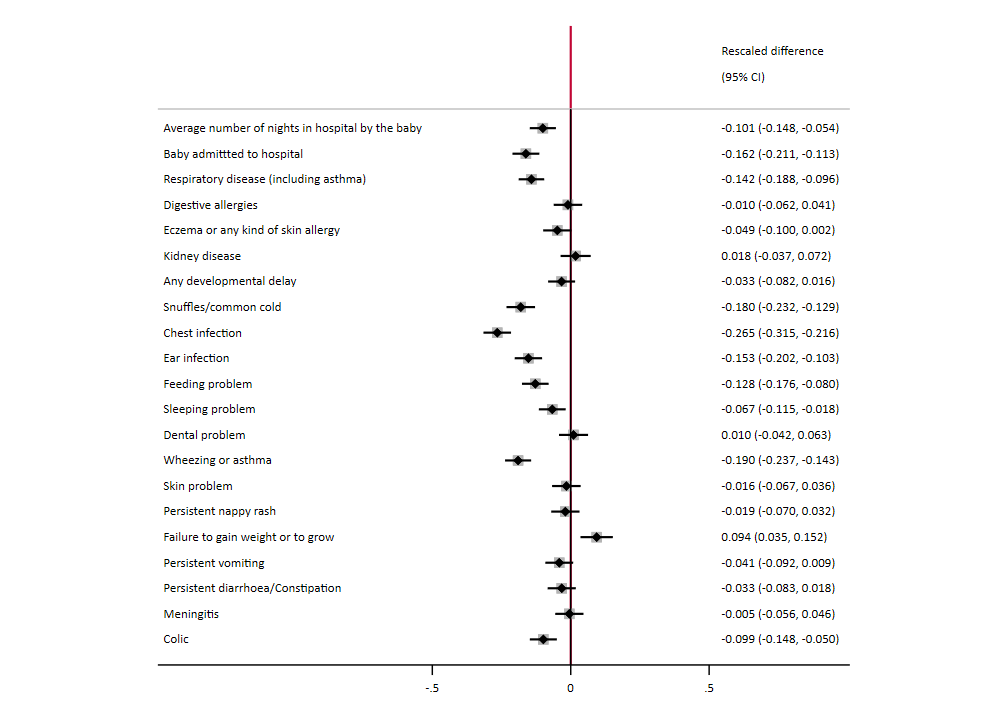


**Figure A5. Unadjusted comparison of outcomes for BF versus non-BF (SA1) rescaled by non-BF standard deviation**


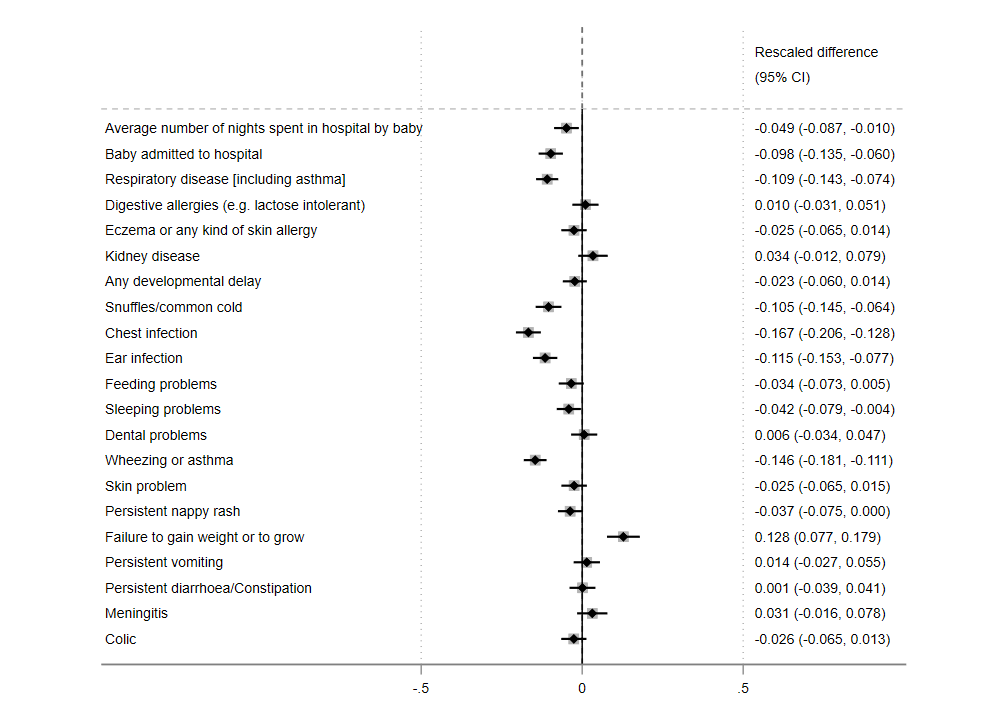


**Figure A6. Unadjusted comparison of outcomes for EBF versus non-EBF (SA2) rescaled by non-BF standard deviation**


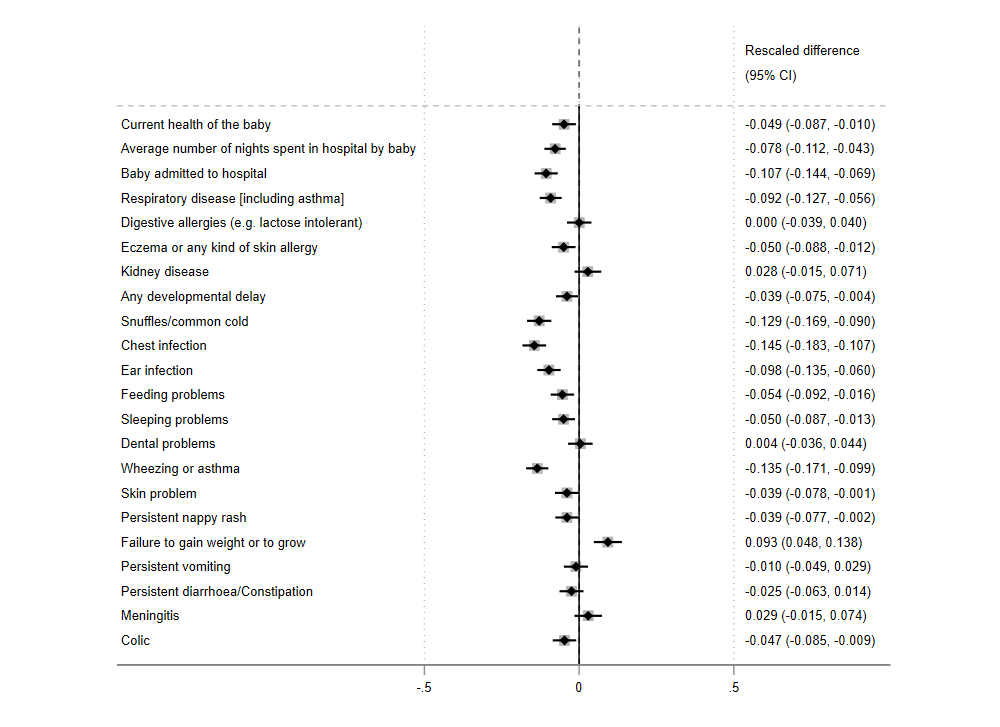


**Figure A7. Unadjusted comparison of outcomes for EBF (90+ days) versus non-EBF (SA3) rescaled by non-EBF standard deviation**


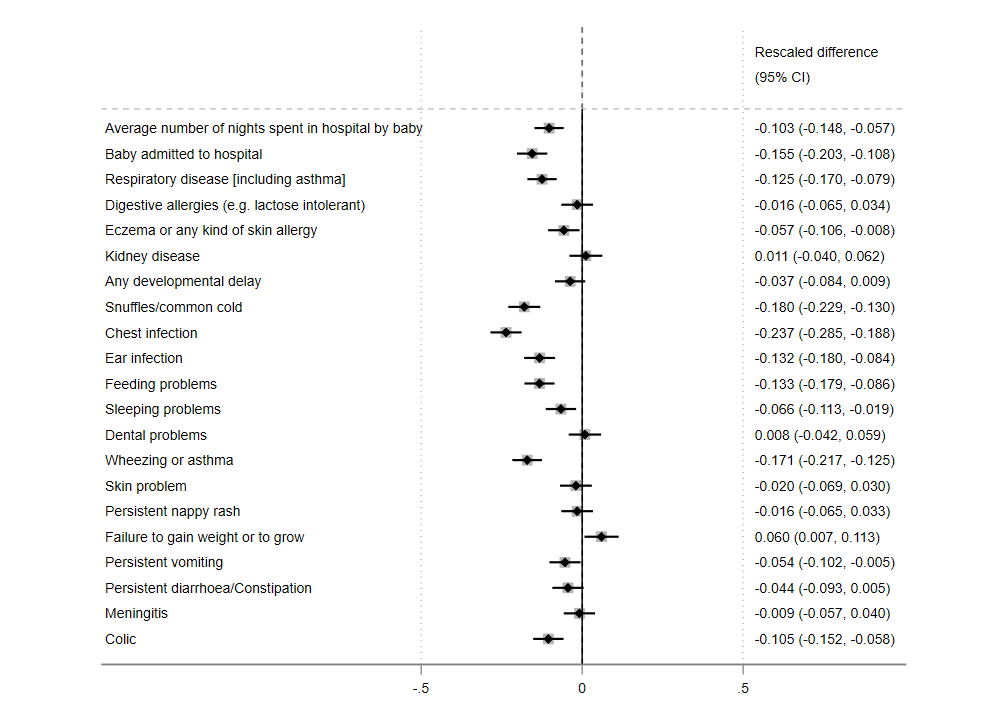


**Figure A8. Propensity score matched comparison of outcomes for EBF (90+ days) versus non-BF rescaled by non-BF standard deviation**


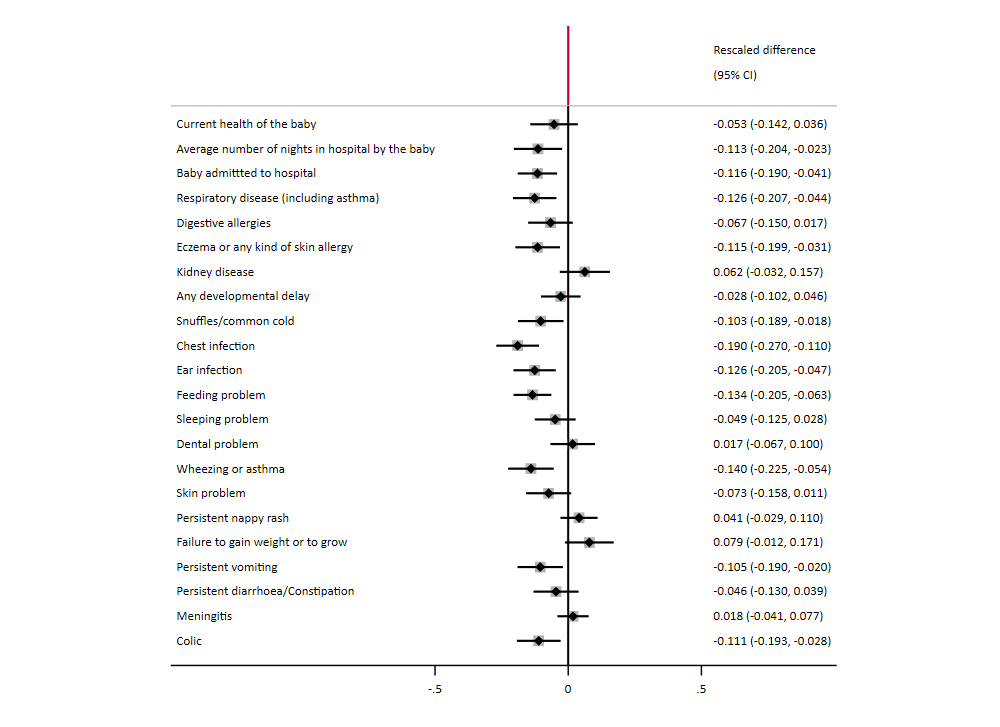


**Figure A9. Propensity score matched comparison of outcomes for BF versus non-BF (SA1) rescaled by non-BF standard deviation**


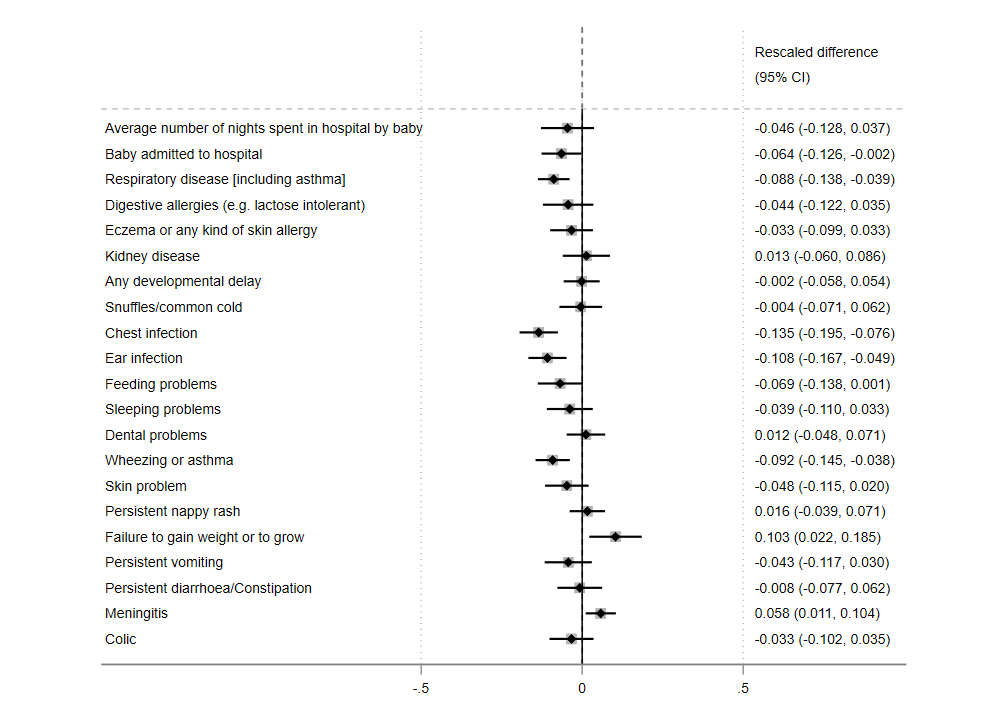


**Figure A10. Propensity score matched comparison of outcomes for EBF versus non-EBF (SA2) rescaled by non-BF standard deviation**


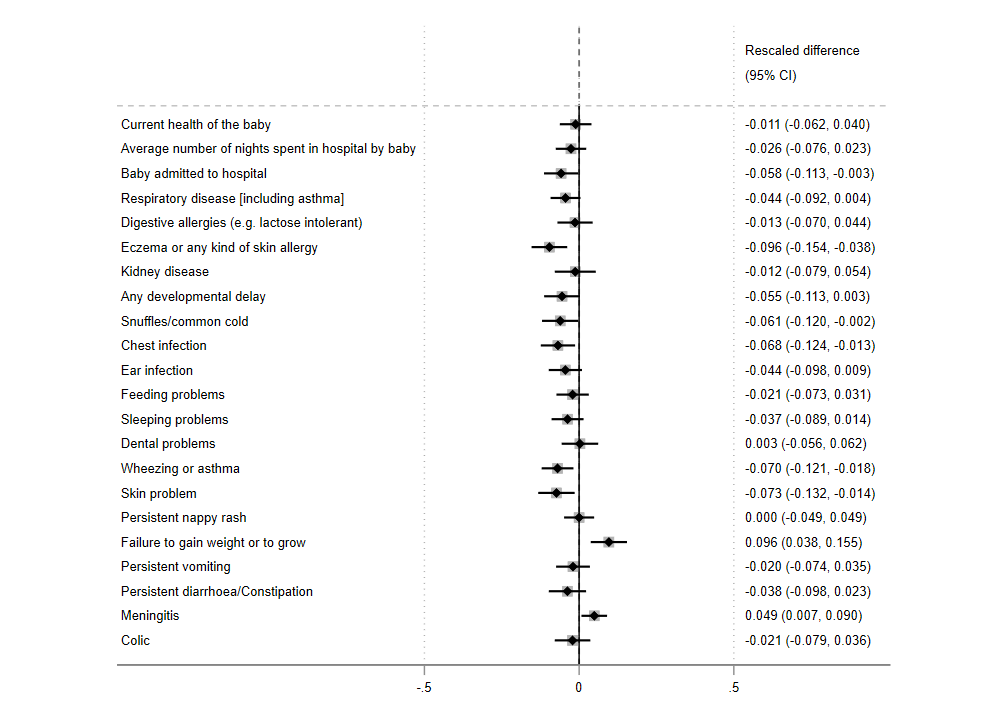


**Figure A11. Propensity score matched comparison of outcomes for EBF (90+ days) versus non-EBF (SA3) rescaled by non-EBF standard deviation**


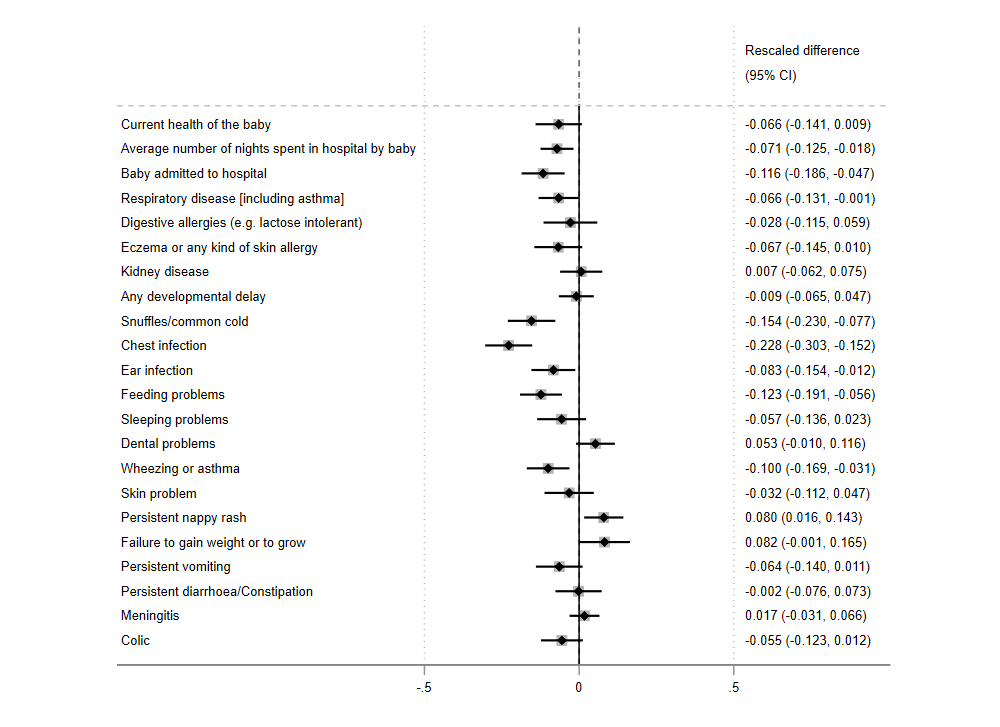


**Figure A12. Flow chart for analysis sample.**

Questionnaire Results Assessed for eligibility

(n = 11,134)

(70.2% response rate)

# Enrollment

Excluded (n = 1,255)

Absence of examined variables

Missingness of data as per Table A1

Eligible for Analysis

(n=9,879)

#

Non-Breastfed

(Non-BF) Group

(n=3,987)

Allocation

Exclusive Breastfed for 90 days

(EBF90) Group

(n=2,212)

# Analysis

Analyzed (n = 2,212)

Entropy Balancing

Excluded from analysis

(n =0)

Analyzed (n =3,987)

Entropy Balancing

Excluded from analysis

(n =0)
