# Supplementary material for: Exploring the relationship between breastfeeding and the incidence of infant illnesses in Ireland: evidence from a nationally representative prospective cohort study
Source: BMC Public Health. 2023 Jan 20;23:140. doi: 10.1186/s12889-023-15045-8 (PMC9854149; doi:10.1186/s12889-023-15045-8)
Supplement: Supplementary file 3 — Additional file 3. [file 12889_2023_15045_MOESM3_ESM.docx]

**Online Appendix:**

## A1 Missing data

## Household equivalised income was missing for 864 infants, with a further 198 missing information on how antenatal care was provided, 104 missing weight at birth and 89 missing information for at least one of the remaining variables. As can be seen in Table A1 in the appendix, the rate of missingness was similar across our comparison groups, suggesting our assumption that data is ‘missing at random’ is plausible in this context. More precisely, while there may be a mechanism influencing missingness, since it appears to affect our comparison groups in the same manner, it should not influence the differences in outcomes between groups which is our primary interest here.

## A2 Entropy Balancing

As with PSM and IPW, entropy balancing requires the assumption that unobserved cofounders are balanced between groups i.e., it assumes conditional independence of treatment and outcomes conditional on observed covariates. Entropy balancing seeks to maximise the entropy of the weights ($w_{i}log(w_{i})$) subject to the weights being positive and summing to 1, while balancing the moments of the covariate distributions:

Eq. (A.1)

$$\max_{w} -\sum_{D_{i}=0} w_{i}log(w_{i}) subject to:$$

$$\sum_{D_{i}=0} w_{i}c_{j} \left( X_{i} \right)=\frac{1}{n_{1}}\sum_{D_{i}=1} c_{j} \left( X_{i} \right),$$

$$\sum_{D_{i}=0} w_{i}=1$$

$$w_{i}>0$$

$$for j=1,..,p and i=1,\ldots,n$$

where $c_{j} \left( X_{i} \right)$ indicates the *p*^th^ moment function of a given covariate, *X_i_*. After finding the optimal weights, *w^EB^*, the average treatment effect on the treated (ATT) can be estimated as:

$$\hat{\tau}=\sum_{D_{i}=1} \frac{Y_{i}}{n_{1}}-\sum_{D_{i}=0} w_{i}^{EB}Y_{i}$$

## A3 Sensitivity Analyses

To test the sensitivity of our results to unobserved confounders, we re-estimate the effects using propensity score matching ^19^ and calculate Rosenbaum bounds ^43^ for the average treatment effect on the treated in the presence of unobserved heterogeneity (hidden bias) between treatment and control cases. We use the user-written Stata command *mhbounds* ^44^ for binary outcomes and *rbounds* ^45^ for continuous. *mhbounds* calculates Mantel-Haenszel tests statistics that give bound estimates of significance levels at given levels of hidden bias under the assumption of either systematic over- or underestimation of treatment effects. *rbounds* calculates Wilcoxon signed rank tests that give upper and lower bound estimates of significance levels at given levels of hidden bias.

We assess the sensitivity of our PS matched analyses to unobserved confounders that would bias the estimated difference positively for outcomes where we found a positive difference between groups (e.g., failure to grow) or negatively where we found a negative difference (e.g., admission to hospital) based on propensity score matched results. We find that propensity score matched results would be sensitive to the existence of unobserved confounders that increase the probability of the infant being EBF90days, EBF, or BF by between 10% and 30% depending on the outcome and comparison being considered. This sensitivity may in part reflect the fact that these estimated differences tended to be less statistically significant to begin with than our entropy balanced results. Nonetheless they suggest a degree of caution is warranted in interpreting the results as causal rather than associative.

## A4 Multiple testing

A further possible concern is multiple testing, since we consider 22 outcomes. We apply the Šidák (1967) ^46^ and the more conservative Bonferroni (1936) ^47^ corrections and find that both corrections agreed for all comparisons. Of the 16 statistically significant differences for the EBF90days versus non-BF comparison, 11 remained statistically significant after correction, the exceptions being the current health, vomiting, skin problems, sleeping problems and nappy rash. For the BF versus non-BF comparison, 6 out of the 11 statistically significant differences, remained statistically significant, the exceptions being the average number of nights spent in hospital, eczema, skin problems, sleeping problems and meningitis. For the EBF versus non-EBF comparison 9 out of 12 statistically significant differences continued to be significant, the exception being the average number of nights spent in hospital, sleeping problems and feeding problems. For the EBF90days versus non-EBF comparison 11 out of 15 differences remained significant after applying the correction, with failure to grow, current health, vomiting and skin problems becoming insignificant after the correction.
